# Zero-Shot Vertebral Instance Segmentation on DICOM Spine Radiographs Using Promptable Segment Anything Models

**DOI:** 10.3390/jcm15052042

**Published:** 2026-03-07

**Authors:** Alexander Sieradzki, Kamil Koszela, Szymon Koszykowski, Jakub Bednarek, Jarosław Kurek

**Affiliations:** 1 Department of Artificial Intelligence, Institute of Information Technology, Warsaw University of Life Sciences, ul. Nowoursynowska 159, 02-776 Warsaw, Poland; 2 Department of Musculoskeletal Disorders, National Institute of Geriatrics, Rheumatology and Rehabilitation, ul Spartańska 1, 02-637 Warsaw, Poland; 3 Faculty of Medicine, Medical University of Lodz, Kościuszki 4, 90-419 Łódź, Poland

**Keywords:** vertebral instance segmentation, DICOM radiographs, zero-shot segmentation, promptable segmentation, foundation models, Segment Anything Model, SAM2, MedSAM, medical image analysis

## Abstract

**Background:** Accurate vertebral instance segmentation on full-spine radiographs is essential for spinal parameter assessment, but supervised methods require costly instance-level annotations and may be sensitive to domain shift. **Methods:** We investigated whether promptable segmentation foundation models can generalize zero-shot to raw DICOM spine radiographs without task-specific training. We evaluated SAM-ViT-Huge, SAM2-Hiera-Large, and MedSAM-ViT-Base on 144 full-spine radiographs with 1309 annotated vertebral masks using a standardized pipeline for DICOM decoding, intensity normalization, automatic prompt generation, and instance-level evaluation. For each prompt, models produced three candidate masks. Performance was reported under an oracle protocol selecting the candidate with the highest IoU against ground truth and a model-score protocol selecting the candidate with the highest predicted IoU. Metrics included IoU, Dice, precision, recall, ASSD, and HD95. **Results:** The best configuration was SAM-ViT-Huge with rectangle prompting, reaching a mean IoU/Dice of 0.782/0.870 under oracle selection and 0.737/0.837 under model-score selection. SAM2-Hiera-Large with rectangle prompting followed (0.744/0.848 oracle; 0.699/0.815 model-score), ahead of MedSAM-ViT-Base (0.599/0.737 oracle; 0.387/0.499 model-score). Point prompting yielded consistently low overlap (IoU 0.224–0.319; Dice 0.276–0.414) despite high recall, indicating systematic over-segmentation and large boundary errors. **Conclusions:** Zero-shot vertebral instance segmentation on raw DICOM spine radiographs is feasible with promptable foundation models when prompts sufficiently constrain target extent. Rectangle prompting is clearly more effective than point prompting in this setting.

## 1. Introduction

Quantitative analysis of full-spine radiographs remains central to diagnosis of spine disorders, treatment planning and longitudinal follow-up. Beyond visual assessment, routine workflows increasingly rely on reproducible measurements and structured anatomical cues, where vertebral delineation can support downstream tasks such as vertebra localization, indexing and geometry-based measurements. However, vertebral instance segmentation on radiographs is intrinsically challenging. Projection imaging compresses 3D anatomy into a 2D plane, leading to overlaps, foreshortening and weak or partially missing boundaries; radiographs may also exhibit noise, low contrast and acquisition-dependent artifacts. These factors, coupled with large inter-patient and inter-level anatomical variability, make reliable vertebral delineation difficult even for modern learning-based approaches [1].

Most high-performing vertebra segmentation systems have been developed and validated primarily in CT and MRI, where volumetric context and clearer tissue interfaces facilitate delineation. Large curated datasets and benchmarks have enabled systematic progress, including multi-center evaluations and studies focusing on anatomical variations [2,3]. Methodologically, supervised pipelines often combine detection/localization with segmentation and labeling and can reach strong performance when trained on sufficiently large and representative cohorts [4]. In contrast, radiographs—despite being widely available and clinically important—remain less covered by public instance-level annotations and are more prone to domain shift across devices, protocols and patient populations. While dedicated deep-learning approaches for spine X-ray segmentation and labeling have been proposed, they still largely depend on annotated training data and may require careful dataset curation to maintain robustness [5].

The emergence of foundation models has introduced an alternative paradigm for medical image segmentation: promptable, general-purpose models that can be applied in a zero-shot manner. A prominent line of work evaluates the Segment Anything Model (SAM) family in medical imaging, showing that out-of-the-box promptable segmentation can be feasible for certain tasks, but that performance strongly depends on modality, anatomy and prompting strategy [6,7]. Beyond technical benchmarks, clinical-oriented studies indicate that SAM-style models can provide practically useful segmentations in specific scenarios (e.g., brain tumor delineation in MRI for radiotherapy planning), yet often require careful prompt design, quality control and occasionally domain-specific adaptation to achieve reliable boundary accuracy [8]. To mitigate medical-domain appearance shifts, specialized variants and adaptations have been explored, including MedSAM-style models and SAM-compatible fine-tuning recipes, with evidence that medical adaptation can improve robustness in some spine-related settings while still exhibiting modality- and prompt-dependent behavior [9].

Recently, the SAM ecosystem has expanded with newer backbones and interfaces, including SAM2-style models designed to improve efficiency and generalization across images (and originally videos), motivating early investigations of their applicability to 2D/3D medical segmentation. Initial medical studies report that SAM2 can be deployed for a range of anatomical structures, but limitations persist in low-contrast boundaries and dense-instance settings [10]. Importantly for spine applications, analyses focusing on vertebral targets highlight that the success of learning-free segmentation depends not only on the backbone but also on how the target instance is specified, particularly when multiple vertebrae with highly repetitive appearance occupy the field of view [11].

Prompt engineering therefore emerges as a key determinant of zero-shot performance. Across medical SAM studies, bounding-box prompts are frequently more reliable than single-point prompts because they provide explicit extent information and reduce ambiguity in crowded anatomical scenes [12]. In parallel, automated or training-free prompt placement strategies aim to reduce manual effort and improve consistency, for example by propagating prompts in structured imaging scenarios [13]. Other lines of work incorporate stronger priors through pseudo-mask prompts or external guidance; for spine imaging, multi-atlas or pseudo-label guidance has been used to steer SAM-like models toward anatomically plausible solutions without manual mask annotation [14]. Finally, when strict zero-shot performance is insufficient, parameter-efficient fine-tuning (PEFT) approaches have been proposed to adapt SAM-like models with limited labeled data, providing a practical compromise between out-of-the-box generalization and fully supervised retraining [15]. Complementary to prompting and adaptation, integrating anatomical priors (e.g., statistical or implicit shape models, deformable templates) remains a well-established direction for improving robustness under weak boundaries and high variability and can be viewed as synergistic with promptable segmentation in challenging spine imaging settings [16,17].

### 1.1. Related Work and Technical Context

#### 1.1.1. Supervised Vertebral Segmentation and Benchmarks

Most vertebral segmentation literature has been developed for CT and MRI, where volumetric context and clearer tissue interfaces simplify delineation compared to projection radiography. Early and representative MRI pipelines include vertebral body segmentation in wide-range routine spine MRI [18] and hybrid methods that combine CNNs with geometric optimization for whole-spine MRI [19]. In CT, strong supervised baselines have been reported using iterative fully convolutional networks for joint segmentation and identification [4], cascaded CNN pipelines for segmentation/localization/identification [20] and hybrid learning–clustering schemes [21]. Large public datasets and benchmarks (e.g., multi-detector CT labeling/segmentation challenges and multi-vendor datasets with anatomical variations) have enabled systematic evaluation and generalization studies [2,3]. Recent workflows continue to improve robustness and automation, for example through two-phase multi-class semantic and instance segmentation on T2-weighted MR images [22], multi-stage networks for vertebra identification driven by mask prompts [23], semantics–instance interactive learning in CT [24] and fully automated segmentation/labeling pipelines targeting clinical deployment [25]. Related spine-region segmentation work also covers intervertebral disc analysis and joint vertebra–disc segmentation designs [26,27], as well as broader biomedical segmentation architectures such as multi-scale attention-based networks [28].

Despite this progress, radiographs remain comparatively under-served by large instance-level annotations and reproducible benchmarks, even though they are central for spinal parameters assessment. Dedicated X-ray approaches exist, including CNN-based lumbar spine X-ray segmentation and labeling [5] and hybrid U-Net/FPN variants for spine segmentation [29]. A comprehensive radiography-focused review highlights that projection artifacts, weak boundaries and acquisition variability complicate vertebral localization and measurement [1]. Additional sources of variability include demographic and anatomical differences (e.g., reported differences in lumbar pedicle angles across ethnic groups), which motivate robust modeling across populations [30]. Overall methodological context is provided by broader surveys of deep learning in medical image analysis, including trends and open challenges relevant to segmentation and generalization [31].

#### 1.1.2. Anatomical Priors, Shape Models and Synthetic Data

Because radiographs often exhibit missing/ambiguous boundaries, multiple lines of work incorporate stronger geometric or anatomical priors. Classical statistical shape modeling and correspondence estimation provide one avenue to encode plausible variability, including robust kernel PCA shape modeling under erroneous annotations [32] and inter-subject correspondence computation without explicit organ segmentation [33]. More recent learning-based priors include deep implicit statistical shape models for lumbar vertebrae delineation [16] and structural descriptors that enforce low-rank shape consistency across vertebrae [34]. Decomposition and template-based formulations provide complementary options, such as skeletonization-driven decomposition into anatomical regions [35] and differentiable appearance modeling of a deformable spine template to achieve training-free vertebral segmentation [17]. Parallel efforts explore modern sequence/state-space backbones and explicit shape priors in spinal segmentation, including residual visual Mamba layers [36] and hierarchical Mamba-based spinal segmentation frameworks [37]. Finally, synthetic or virtual case generation (e.g., creating virtual spine cases) has been investigated as a way to expand variability and stress-test segmentation and measurement pipelines [38]. These developments connect to broader frameworks that emphasize geometric scene understanding for surgical data science and digital twins, where accurate anatomical segmentation is a core enabling capability [39].

#### 1.1.3. Promptable Foundation Models and Zero-Shot Medical Segmentation

The emergence of promptable foundation models has shifted attention toward training-free or minimally supervised segmentation. SAM-style models have been systematically evaluated in medical imaging, demonstrating both opportunities and limitations that depend on modality and prompting strategy [6,7]. Empirical assessments on specific modalities (e.g., brain tumor MRI and digital pathology) further highlight that zero-shot performance can be useful but requires careful interaction design and quality control [40,41], with clinical studies reporting favorable but imperfect accuracy in radiotherapy-relevant settings [8]. Several works propose general improvements or practical guidelines for enhancing SAM behavior in medical images [42,43]. For spine imaging, comparisons between SAM and medically adapted variants such as MedSAM indicate modality-dependent trade-offs on lumbar spine MRI [9].

The SAM ecosystem has expanded with SAM2, motivating early comparisons between SAM and SAM2 in medical segmentation [44] and studies applying SAM2 to 2D/3D medical data [10]. Clinical/clinical-adjacent investigations also analyze learning-free vertebral segmentation with metastatic lesions and characterize factors affecting SAM2 performance [11], while other studies adapt SAM2’s tracking capabilities for zero-shot 3D segmentation in CT [45]. For radiography specifically, recent work leverages foundation-model concepts to target automatic spine X-ray segmentation [46], aligning with the broader question of how well promptable backbones transfer to projection imaging.

#### 1.1.4. Prompt Engineering and Automated Prompting

A consistent conclusion across promptable segmentation literature is that prompt design strongly shapes outcomes. Systematic studies quantify how different prompts affect SAM performance in challenging medical settings, often finding that box- or region-constraining prompts outperform sparse points [12,47]. Overviews of prompt engineering in medical segmentation synthesize these observations and outline interaction paradigms and failure modes [48]. Beyond manual prompting, multiple approaches pursue automated prompt generation and placement, including training-free prompt propagation in 3D bone CT [13] and fully automated prompting pipelines for 3D multi-organ segmentation [49]. Other work introduces stronger priors into the prompt itself, for instance by using registration-enabled prompt engineering with reference images [50] or one-shot, reference-guided, training-free point prompting for segmenting arbitrary tissues [51]. Prompt robustness has also been studied beyond standard imaging, such as variational prompting in non-visible spectrum imagery [52], providing transferable insights into prompt uncertainty and ambiguity.

Prompting can be further enriched via multi-modal guidance and pseudo-mask cues. Text–image integration has been explored through CLIP-augmented promptable models, e.g., MedCLIP-SAM [53] and SAM+CLIP cascades for test-time adaptation [54]. Similarly, atlas- or pseudo-mask-guided prompting has been proposed for spine segmentation without manual mask prompts [14]. At the other end of the interaction spectrum, “one-prompt” formulations aim to generalize segmentation from a single user signal across tasks and modalities [55] and semantic-integration variants adapt SAM toward zero-shot medical semantic segmentation [56]. In addition, methods that explicitly target improved dichotomous (binary) segmentation quality under the SAM interface continue to appear [57].

#### 1.1.5. Adaptation, 3D Extensions and Parameter-Efficient Fine-Tuning

When strict zero-shot performance is insufficient, a large body of work explores how to adapt SAM-like models efficiently. 3D extensions and adaptation recipes include volumetric SAM formulations [58], modality-agnostic adaptation for 3D medical segmentation [59] and Mamba-based efficient adaptations such as tri-plane designs for 3D data [60]. Training-time or inference-time strategies that add minimal parameters have been proposed for 2.5D/3D adaptation [61]. More broadly, parameter-efficient fine-tuning (PEFT) has been emphasized as a practical compromise for medical image analysis under limited labels, with analyses of missed opportunities [62], orchestration strategies for SAM adaptation [63] and SAM-to-medical transfer via PEFT [15]. Related PEFT work on transformer backbones is also being explored in neuroimaging analysis [64] and in no-reference image quality assessment, which is relevant for quality-aware deployment on heterogeneous radiographs [65]. Finally, boundary-aware supervision strategies in other segmentation domains (e.g., cardiac structures) provide transferable ideas for reducing contour errors in weak-boundary settings [66].

Despite this rapid progress, evidence on raw DICOM spine radiographs characterized by large field-of-view, multiple adjacent vertebral instances and substantial projection artifacts—remains limited compared to CT/MRI studies. This work addresses the gap via a pilot benchmark of zero-shot vertebral instance segmentation on full-spine DICOM radiographs using promptable segmentation foundation models. We evaluate three representative backbones (sam-vit-huge, sam2-hiera-large and medsam-vit-base) under a fully standardized pipeline for DICOM decoding and intensity normalization, automatic prompt generation (single centroid point vs. tight bounding box) and instance-level metric computation (overlap and boundary metrics). By isolating the effect of prompt type and model choice in a parameter-free setting, the study aims to clarify (i) how far current promptable models generalize to spine radiographs out-of-the-box and (ii) which prompting strategy yields reliable instance-level vertebral masks without interactive refinement.

The remainder of the paper is organized as follows. Section 2.1 describes the evaluation dataset and annotation protocol. Section 3.1 details the compared foundation models and the standardized inference pipeline, including prompt generation and mask selection. Section Results reports quantitative and qualitative outcomes across prompting strategies and backbones, followed by a discussion of failure modes, practical implications and limitations in Section Discussion.

## 2. Materials and Methods

### 2.1. Dataset Description

The experimental dataset consists of spine radiographs acquired for spinal parameters assessment and stored in the DICOM standard [67].

Each radiograph is paired with a JSON annotation file that encodes instance-level vertebral delineations. For the purpose of quantitative evaluation, the annotations were rasterized into binary masks at the native image resolution, yielding one ground-truth mask per vertebra. Because this study focuses on zero-shot segmentation, the dataset was used exclusively for evaluation and no images were used for model fine-tuning.

Table 1 summarizes the dataset size and annotation density. On average, each radiograph contains multiple annotated vertebrae (median 10, range 2–16), enabling instance-level evaluation across a broad spectrum of anatomical coverage. Table 2 reports the distribution of native image resolutions and annotation sizes.

Table 1 provides an overview of the dataset size and annotation density used in this study. The dataset consists of 144 unique spine radiographs acquired in the DICOM format and annotated with a total of 1309 instance-level vertebral masks. Each radiograph contains multiple annotated vertebrae, with an average of 9.09 vertebral instances per image and a standard deviation of 4.26. The number of annotated vertebrae per radiograph ranges from 2 to 16, indicating substantial variability in anatomical coverage. This variability reflects differences in image framing, spinal region visibility and spine disorders severity and enables a robust instance-level evaluation of vertebral segmentation performance across heterogeneous clinical cases.

Table 2 summarizes the spatial characteristics of the radiographs and the corresponding vertebral annotations. The images exhibit heterogeneous native resolutions, with a mean height of 3404 pixels and a mean width of 2575 pixels, highlighting differences in acquisition protocols and imaging equipment. The reported median values and interquartile ranges further confirm the broad distribution of image dimensions across the dataset.

Vertebral annotation sizes, expressed as mask areas in squared pixels (px^2^), show considerable variability. The mean vertebral mask area equals 38,837 px^2^, with a large standard deviation of 28,449 px^2^, indicating the presence of both small and large anatomical structures. This variability arises from differences in vertebral level, projection geometry and spinal curvature and poses a non-trivial challenge for segmentation methods. The reported statistics emphasize the need for segmentation models that are robust to scale variation and resolution heterogeneity.

### 2.2. Qualitative Examples

**Figure 1:**
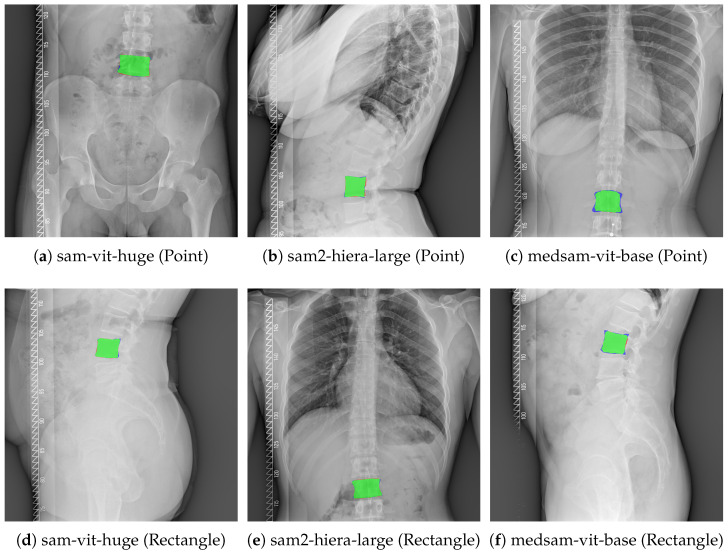
Representative qualitative examples of vertebral segmentation for the six evaluated configurations (SAM-ViT-Huge, SAM2-Hiera-Large and MedSAM-ViT-Base; each under point and rectangle prompting). Colors indicate a difference overlay relative to the ground truth: green—true positives (overlap), red—false positives, blue—false negatives.

To complement the quantitative results in Table 3, Figure 1 presents representative qualitative examples of zero-shot vertebral segmentation for all six evaluated configurations (three backbones × two prompt types). Each panel shows a difference overlay between the predicted mask and the ground-truth mask: true positive overlap is shown in green, false positives in red and false negatives in blue. The examples are shown for illustration of typical boundary deviations observed under each configuration.

## 3. Methods

This study was designed as a pilot feasibility assessment of promptable foundation models for vertebral segmentation on spine radiographs in a strictly zero-shot setting. The primary aim was to quantify how far such models can generalize to DICOM spine radiographs without any task-specific training, rather than to develop an optimized, clinically validated segmentation system.

In the context of this work, zero-shot segmentation refers to applying pre-trained segmentation models directly to a new target domain and task without updating model parameters. Accordingly, no images from the evaluated dataset were used for fine-tuning, calibration, or any form of supervised adaptation and the publicly available model checkpoints were used as-is. The segmentation output is controlled exclusively through a prompt, i.e., a sparse spatial cue that specifies the target structure and initiates mask generation.

The zero-shot approach used here is characterized by the following properties: (i) parameter-free inference on the target dataset (no gradient-based optimization on study images); (ii) reliance on general visual representations learned during large-scale pre-training, which must transfer to radiographic appearance despite domain shift; (iii) prompt-conditioned instance segmentation, where the user (or an automated procedure) provides minimal information to indicate which structure should be segmented; (iv) sensitivity to prompt informativeness and ambiguity, which is particularly relevant in radiographs containing multiple anatomically similar structures in close proximity.

Because this is a pilot evaluation, we emphasize that the reported results primarily reflect the feasibility and limitations of out-of-the-box generalization under controlled prompting. The experimental protocol was therefore intentionally standardized: prompts were generated automatically from the reference annotations (point at centroid or tight bounding box) to eliminate inter-operator variability and to isolate the effect of prompt type and model choice. This design enables reproducible, model-to-model comparisons, while still reflecting the core constraint of the zero-shot paradigm, namely the absence of any training on the target cohort.

### 3.1. Segmentation Foundation Models

In this study we evaluated three promptable segmentation foundation models that can generate binary object masks conditioned on user prompts such as points and bounding boxes. All models were used in a zero-shot setting, without any fine-tuning on the target radiographs. To ensure a fair comparison, the same image pre-processing, prompt generation procedure, multi-mask handling and metric computation were applied across all evaluated backbones.

#### 3.1.1. SAM-ViT-Huge

The Segment Anything Model (SAM) is a general-purpose foundation model for promptable segmentation designed to generalize across diverse visual domains and object categories [68]. It formulates segmentation as a prompt-conditioned prediction problem: given an image and a prompt that specifies the target (e.g., a foreground point or a bounding box), the model outputs one or more candidate masks corresponding to the prompted object. The SAM architecture is composed of three key components: (i) an image encoder that maps the input image into a latent embedding space, (ii) a prompt encoder that converts user prompts (points, boxes and optional mask inputs) into prompt embeddings and (iii) a mask decoder that fuses image and prompt embeddings to produce segmentation masks and mask-quality estimates.

In our benchmark we used the facebook/sam-vit-huge checkpoint, which corresponds to the largest SAM image-encoder variant based on a Vision Transformer backbone (ViT-H). This model variant represents the highest-capacity SAM configuration commonly used for zero-shot segmentation and interactive annotation workflows. In the standard SAM inference mode, the model may return multiple candidate masks for a single prompt together with a predicted mask-quality score (often interpreted as an estimated IoU), which can be used to select the most plausible output when ground truth is not available [68].

Within our pipeline, SAM inference was performed via the Hugging Face implementation (SamModel and SamProcessor). For computational efficiency, image embeddings were computed once per radiograph and reused for all vertebral instances from that image, ensuring identical encoder outputs regardless of the number of vertebrae annotated in the same radiograph.

#### 3.1.2. MedSAM-ViT-Base

MedSAM is a medical imaging foundation model derived from the SAM paradigm, designed to improve segmentation robustness under medical-domain appearance shifts (e.g., modality differences, weak boundaries, low contrast and domain-specific textures) [69]. Unlike the original SAM, which is primarily trained on natural images, MedSAM is obtained by fine-tuning the SAM-style promptable segmentation architecture on a large-scale curated medical segmentation corpus. According to the MedSAM publication, the model is developed using a dataset containing 1,570,263 image–mask pairs spanning 10 imaging modalities and more than 30 cancer types, with extensive internal and external validation across many segmentation tasks [69]. This training strategy aims to provide a universal promptable model that can be applied across heterogeneous medical imaging scenarios.

From an architectural perspective, MedSAM follows the same modular design as SAM, relying on an image encoder, a prompt encoder and a mask decoder. The model is explicitly designed for promptable segmentation, where user inputs (commonly bounding boxes and/or points) define the target region of interest. MedSAM emphasizes the practical use of bounding-box prompts as an efficient and unambiguous way to specify targets, while still supporting point prompts. The MedSAM framework is also compatible with applying 2D promptable segmentation slice-wise to 3D acquisitions, enabling broad applicability across modalities.

In our evaluation we used the wanglab/medsam-vit-base checkpoint (ViT-Base backbone). Inference was performed using the same Hugging Face SAM-compatible API (SamModel/SamProcessor), which allowed us to keep the pre-processing and prompting interface consistent with the sam-vit-huge configuration. This made it possible to isolate the effect of medical-domain adaptation (MedSAM) from prompt type and evaluation protocol.

#### 3.1.3. SAM2-Hiera-Large

SAM2 extends the Segment Anything concept from static images to both images and videos, aiming to provide a unified foundation model for promptable visual segmentation with improved efficiency and robustness [70]. A key design element of SAM2 is the use of a transformer architecture equipped with streaming memory, which enables real-time processing and propagation of segmentation information across video frames. Although our dataset consists of single-frame radiographs (i.e., the image setting), the same promptable interface is retained: point and bounding-box prompts can be used to generate object masks and the model can output multiple candidate masks per prompt.

In this benchmark we used the facebook/sam2-hiera-large checkpoint, which corresponds to the Hiera-Large backbone variant. The official SAM2 release provides several checkpoint sizes; the Hiera-Large configuration represents a high-capacity option within the SAM2 family and is substantially smaller than the ViT-H SAM model, reflecting architectural and efficiency differences between the SAM and SAM2 design choices. For SAM2 inference we used the official SAM2ImagePredictor interface. As in the other models, segmentation was performed under both point and rectangle prompting and the multi-mask output mode was enabled to obtain a set of candidate masks per prompt for subsequent selection under our evaluation protocol.

Overall, the three evaluated backbones represent complementary design points: sam-vit-huge provides a high-capacity generalist SAM baseline; medsam-vit-base tests the impact of medical-domain adaptation within a SAM-compatible architecture; and sam2-hiera-large evaluates a newer promptable segmentation foundation model designed to generalize across images and videos with an updated backbone and memory-based architecture.

### 3.2. Benchmarking Pipeline and Metric Computation

All experiments were conducted using an automated benchmarking pipeline implemented in Python 3.11.14.

The pipeline evaluates zero-shot vertebral segmentation in a fully standardized manner, ensuring identical pre-processing, prompting strategy and metric computation across all models and cases. For each DICOM radiograph, every annotated vertebra (instance mask) is treated as an independent evaluation sample and all metrics are computed at the native image resolution.

#### 3.2.1. Image Loading and Pre-Processing

Radiographs were loaded directly from the DICOM standard [67]. A robust loading procedure was used: the pipeline first attempted to decode images using an internal DICOM utility function; if this failed, it fell back to header-driven decoding with pydicom [71]. Pixel intensities were converted to floating point, optionally rescaled using the DICOM tags RescaleSlope and RescaleIntercept and corrected for MONOCHROME1 photometric interpretation by intensity inversion [67]. We considered intensity windowing based on DICOM display parameters (WindowCenter/WindowWidth) or a VOI LUT to enhance bone contrast; however, because radiographic pixel values are not calibrated to a universal physical scale and windowing metadata can be vendor- and preset-dependent, we did not apply a fixed “bone window” in this benchmark. Instead, we used per-image min–max normalization to preserve the full available dynamic range in a reproducible, model-agnostic manner. Min–max normalization was applied per radiograph (not across the dataset), mapping the decoded pixel range to the unit interval [0, 1] prior to conversion to 8-bit. This provides a simple invariance to global brightness/contrast scaling differences caused by acquisition settings and vendor-specific processing. We note that min–max scaling can be influenced by extreme values (e.g., metallic implants, burned-in annotations, or collimation borders); therefore, we retained this transparent baseline and treat more robust alternatives (e.g., percentile-based scaling or histogram standardization) as future work. No additional spatial pre-processing (cropping to the spine, denoising, deblurring, or geometric resampling) was applied before the model-specific processors; resizing and padding were handled by the respective SAM/SAM2 preprocessors to match their expected input resolution.

After per-image min–max normalization, each image was converted to 8-bit grayscale and replicated to a three-channel RGB representation to comply with the input requirements of Segment Anything models. All ground-truth masks were rasterized from the JSON annotations at the native image resolution to prevent artifacts introduced by re-sampling.

#### 3.2.2. Prompt Generation

Two prompting strategies were evaluated: (i) rectangle prompt and (ii) point prompt. For each vertebral instance mask, the rectangle prompt was defined as the tight axis-aligned bounding box enclosing the ground-truth mask. For point prompting, a single positive point was placed at the centroid of the ground-truth mask (in image coordinates). This automatic prompt generation eliminates inter-operator variability and provides a standardized comparison of model behavior under the two prompting modes.

##### Prompting Strategies: Rectangle vs. Point

The Segment Anything Model (SAM) framework supports multiple interaction modalities, referred to as prompts [68], which guide the segmentation process by providing sparse spatial cues. In this study, two fundamentally different prompt types were systematically evaluated: rectangle (bounding-box) prompts and point prompts. Both prompting strategies were generated automatically from the ground-truth annotations to ensure full reproducibility and to eliminate inter-operator variability.

##### Rectangle (Bounding-Box) Prompting

In rectangle prompting, the model is provided with an axis-aligned bounding box that tightly encloses the target object. For each vertebral instance, the rectangle prompt was constructed as the minimal bounding box covering all foreground pixels of the corresponding ground-truth mask. Formally, let $M\subset Z^{2}$ denote the set of pixels belonging to a vertebra. The bounding box *B* is defined as [68]:

(1) $$B=[x_{\min},y_{\min},x_{\max},y_{\max}],$$

where

$$x_{\min}=\min_{(x,y)\in M}x,\;x_{\max}=\max_{(x,y)\in M}x,$$

$$y_{\min}=\min_{(x,y)\in M}y,\;y_{\max}=\max_{(x,y)\in M}y.$$

This rectangle prompt provides the model with explicit information about the spatial extent of the vertebra, constraining the segmentation to a well-defined region of interest. As a result, rectangle prompting strongly restricts the search space and reduces ambiguity caused by surrounding anatomical structures. Within the SAM architecture, the bounding box is encoded as a pair of corner coordinates and injected into the prompt encoder, where it conditions the mask decoder via cross-attention with image embeddings.

##### Point Prompting

In point prompting, the model is guided using one or more sparse point annotations indicating object presence or absence. In this benchmark, a single positive point prompt was used for each vertebral instance. The point was placed at the centroid of the ground-truth mask, computed as [72]:

(2) $$\left(x_{c},y_{c}\right)=\left(\frac{1}{\left|M\right|}\sum_{(x,y)\in M}x,\;\frac{1}{\left|M\right|}\sum_{(x,y)\in M}y\right).$$

This centroid-based point represents a minimal localization cue, indicating only that the target object is present at a specific location, without conveying any information about its spatial extent or shape. The point prompt is encoded as a positive point (label =1) in the SAM prompt encoder and influences the segmentation primarily through local attention around the indicated location.

##### Conceptual Differences Between Prompt Types

Rectangle and point prompting differ fundamentally in the amount of spatial information they provide. Rectangle prompts explicitly define both object location and approximate size, thereby enabling the model to focus on a constrained region and suppress irrelevant structures. In contrast, point prompts provide only a weak localization signal, leaving the model responsible for inferring the object’s full extent from global image context and learned priors.

These differences are particularly relevant in medical radiographs, where multiple anatomically similar structures coexist in close proximity. In such settings, point prompting may lead to over-segmentation or leakage into neighboring vertebrae, while rectangle prompting offers stronger spatial disambiguation. By evaluating both prompting strategies under identical experimental conditions, this study isolates the impact of prompt informativeness on zero-shot vertebral segmentation performance.

##### Prompt Consistency Across Models

Importantly, the same rectangle and point prompts were used across all evaluated models—SAM-ViT-Huge, MedSAM-ViT-Base and SAM2-Hiera-Large. This ensured that any observed performance differences arise from model architecture or training rather than from prompt design. Furthermore, prompt generation relied exclusively on geometric properties of the ground-truth masks and did not involve any manual interaction, making the evaluation fully automated and reproducible.

#### 3.2.3. Model Inference and Multi-Mask Output

Three segmentation backbones were evaluated: SAM-ViT-Huge, MedSAM-ViT-Base and SAM2-Hiera-Large. For transformer-based SAM models (SAM-ViT-Huge and MedSAM-ViT-Base), inference was performed using Hugging Face SamModel and SamProcessor. Image embeddings were computed once per radiograph and reused across all vertebral instances in that image, reducing redundant computation. For SAM2, inference was performed using the official SAM2ImagePredictor API, with the image set once per radiograph. In all configurations, the models produced a multi-mask output consisting of three candidate segmentation masks per prompt.

#### 3.2.4. Mask Selection and Post-Processing

To obtain a single prediction for evaluation, the multi-mask output was reduced to one mask per vertebra. SAM-like models output multiple candidate masks for a single prompt to represent alternative plausible segmentations (e.g., under prompt ambiguity) and to support interactive user choice among candidates. The primary evaluation used an oracle selection protocol, in which the candidate mask that maximized IoU with the ground-truth mask was selected. Oracle selection should therefore be interpreted as an optimistic upper bound (a hypothetical “perfect user” always choosing the best candidate), whereas a non-oracle real-world application must select a mask without access to ground truth. This protocol quantifies the best achievable performance given the prompt and the model’s candidate set and is particularly useful when comparing prompt types under a consistent selection rule.

In addition, the pipeline computed model-selected metrics, in which the candidate mask was selected using the model-provided confidence or predicted IoU score (when available). This second selection mode better reflects realistic deployment, where ground truth is not available at inference time. Both oracle-selected and model-selected results were exported to the output spreadsheets to enable transparent reporting and sensitivity analysis.

As a classical (non-learned) reference, we also evaluated a prompt-only baseline for rectangle prompts, where the bounding box prompt itself is used as a filled mask (all pixels inside the box are labeled as foreground). This provides context for interpreting zero-shot segmentation performance relative to coarse localization alone.

After selection, predicted masks were optionally cleaned using a lightweight morphological post-processing routine (noise removal and mask regularization) to reduce isolated artifacts.

#### 3.2.5. Pixel-Wise Segmentation Metrics

All segmentation metrics were computed at the pixel level by comparing the predicted binary mask with the ground-truth binary mask. For each vertebral instance, the following confusion counts were computed: true positives (TP), false positives (FP), false negatives (FN) and true negatives (TN).

Overlap metrics were computed as [73,74]:

(3) $$\mathrm{IoU}=\frac{TP}{TP+FP+FN},$$

(4) $$\mathrm{Dice}=\frac{2TP}{2TP+FP+FN}.$$

Classification-style metrics were computed as [73]:

(5) $$\mathrm{Precision}=\frac{TP}{TP+FP},\;\mathrm{Recall}=\frac{TP}{TP+FN},\;\mathrm{Specificity}=\frac{TN}{TN+FP},$$

(6) $$\mathrm{Accuracy}=\frac{TP+TN}{TP+FP+FN+TN},\;\mathrm{BalancedAccuracy}=\frac{\mathrm{Recall}+\mathrm{Specificity}}{2},$$

(7) $$F1=\frac{2\;\cdot \;\mathrm{Precision}\;\cdot \;\mathrm{Recall}}{\mathrm{Precision}+\mathrm{Recall}}.$$

Agreement measures were also computed. Matthews correlation coefficient (MCC) was calculated as [75]:

(8) $$\mathrm{MCC}=\frac{TP\;\cdot \;TN-FP\;\cdot \;FN}{\sqrt{\left(TP+FP)(TP+FN)(TN+FP)(TN+FN\right)}+\epsilon },$$

where ϵ is a small constant used for numerical stability. Cohen’s kappa was computed from the observed agreement and expected agreement under independence [76]:

(9) $$\kappa =\frac{p_{o}-p_{e}}{1-p_{e}+\epsilon },$$

with $p_{o}=\left(TP+TN\right)/N$ and $p_{e}$ derived from marginal probabilities of positive/negative predictions and labels.

To characterize scale-related behavior, mask areas were recorded as pixel counts (interpretable as squared pixels, px^2^), including ground-truth area, predicted area, absolute and relative area error and the area ratio between prediction and ground truth.

#### 3.2.6. Surface Distance Metrics

To complement region-based overlap measures, boundary-based surface distance metrics were computed when the necessary numerical routines were available. Binary mask surfaces were extracted using one-step morphological erosion and symmetric surface distances were computed using a Euclidean distance transform [77]. The following metrics were reported: average symmetric surface distance (ASSD), 95th percentile Hausdorff distance (HD95) and maximum Hausdorff distance (HD100) [73,78].

When DICOM pixel spacing metadata (PixelSpacing or ImagerPixelSpacing) was available, distances were expressed in millimeters. Otherwise, distances were reported in pixels.

#### 3.2.7. Aggregation, Uncertainty Estimation and Statistical Comparisons

Metrics were computed for each vertebral instance and then aggregated across the dataset. The main results were reported as mean values across all vertebral instances for each model and prompt type, consistent with the case-level evaluation shown in Table 3.

To quantify uncertainty while accounting for multiple vertebrae per radiograph, we computed 95% confidence intervals for mean metrics using a clustered (radiograph-level) bootstrap [79]. Specifically, we resampled radiographs (image_id) with replacement (B=5000 resamples; fixed random seed) and recomputed instance-weighted means, reporting the 2.5th and 97.5th percentiles.

To assess statistical significance between prompting strategies and between models, we performed two-sided paired Wilcoxon signed-rank tests on per-radiograph mean metrics [80]. *p*-values were adjusted for multiple comparisons using the Holm–Bonferroni procedure across all metrics and pairwise comparisons, with α=0.05.

## 4. Results

Table 3 presents a comprehensive quantitative comparison of zero-shot vertebral segmentation performance across three foundation-model variants—SAM-ViT-Huge, SAM2-Hiera-Large and MedSAM-ViT-Base—evaluated using two prompting strategies: point-based and rectangle-based prompts. All metrics are reported as mean values computed over individual vertebral instances, with 95% bootstrap confidence intervals, enabling a fine-grained assessment of segmentation accuracy and agreement at the instance level.

Because oracle selection is not available in real-world zero-shot deployment, we additionally report a non-oracle (deployable) evaluation using model-score-based mask selection in Table 4; Table 3 should be interpreted as an optimistic upper bound on attainable performance given the candidate set.

Across all evaluated models, rectangle-based prompting consistently yields substantially higher performance than point-based prompting in overlap-based metrics, including Intersection over Union (IoU) and Dice coefficient, as well as in agreement measures such as Matthews correlation coefficient (MCC) and Cohen’s kappa. This finding indicates that providing explicit spatial extent information via bounding boxes is critical for effective zero-shot vertebral segmentation on radiographic images. In contrast, point prompts alone appear insufficient to constrain the segmentation process, resulting in reduced spatial precision.

For the SAM-ViT-Huge model, rectangle prompting achieves the strongest overall performance among all tested configurations. The model attains a mean IoU of 0.782 and a Dice coefficient of 0.870, accompanied by high precision (0.850) and recall (0.906). The high values of MCC (0.874) and Cohen’s kappa (0.870) further indicate a strong agreement between predicted masks and ground-truth annotations. When compared to point prompting, rectangle prompting improves IoU and Dice by 0.517 and 0.559, respectively, representing a substantial relative gain in segmentation quality. Point-based prompting for SAM-ViT-Huge, despite achieving very high recall (0.911), yields markedly lower precision (0.293) and overlap scores, suggesting extensive over-segmentation with large false-positive regions.

A similar trend is observed for the SAM2-Hiera-Large model. Rectangle prompting results in strong segmentation performance, with IoU and Dice values of 0.744 and 0.848, respectively. Notably, this configuration achieves the highest recall across all evaluated methods (0.918), indicating a high sensitivity to vertebral structures. However, the corresponding precision (0.803) is slightly lower than that observed for SAM-ViT-Huge under rectangle prompting, which suggests a more permissive segmentation behavior and a higher propensity for false-positive predictions. Under point prompting, SAM2-Hiera-Large exhibits the same characteristic pattern as SAM-ViT-Huge, with low IoU (0.224) and Dice (0.276) values combined with high recall (0.907), again indicating over-segmentation in the absence of sufficient spatial guidance.

MedSAM-ViT-Base demonstrates overall lower segmentation performance compared with the two general-purpose SAM variants, particularly under rectangle prompting. The model achieves a mean IoU of 0.599 and a Dice coefficient of 0.737, with corresponding MCC and kappa values of 0.745 and 0.736, respectively. While rectangle prompting significantly improves performance relative to point prompting (ΔIoU = 0.280, ΔDice = 0.323), the absolute performance remains lower than that of SAM-ViT-Huge and SAM2-Hiera-Large. Under point prompting, MedSAM-ViT-Base exhibits reduced recall (0.762) compared with the other models, indicating a higher rate of missed vertebral regions. This suggests that, in the studied radiographic domain, medical-domain adaptation alone does not compensate for the reduced capacity of the base model architecture in a zero-shot setting.

Across all evaluated models, point-based prompting consistently results in very high recall values (ranging from 0.762 to 0.911) combined with low precision (ranging from 0.249 to 0.425) and poor overlap-based metrics. This pattern indicates a systematic tendency toward over-segmentation, where large image regions are incorrectly classified as vertebral structures. In contrast, rectangle-based prompting substantially improves spatial specificity, leading to balanced precision–recall trade-offs and markedly higher agreement with ground truth. These results demonstrate that prompt design is a dominant factor influencing zero-shot segmentation performance.

Overall, the findings reported in Table 3 indicate that large, general-purpose foundation models can achieve clinically meaningful vertebral segmentation performance in a zero-shot setting when provided with appropriate spatial prompts. Among the evaluated approaches, SAM-ViT-Huge combined with rectangle prompting provides the most favorable balance between accuracy, agreement and sensitivity, making it the most robust configuration for zero-shot vertebral segmentation on DICOM radiographs in this study.

### 4.1. Oracle vs. Model-Score Mask Selection

To provide a realistic assessment without access to ground truth at inference time, Table 4 reports performance when the candidate mask is selected using the model-provided confidence (predicted IoU). As expected, model-score-based selection reduces performance relative to the oracle upper bound in Table 3. The gap is modest for rectangle prompting in SAM-ViT-Huge and SAM2-Hiera-Large (ΔIoU ≈ 0.045 in both cases), but substantially larger for MedSAM-ViT-Base under rectangle prompting (ΔIoU = 0.211). For point prompting, model-score selection yields low overlap across all models (mean IoU 0.131–0.222) despite very high recall (0.724–0.955), consistent with systematic over-segmentation under underconstrained prompts.

Paired statistical testing (Section 3.2.7) confirmed that rectangle prompting significantly outperforms point prompting for all models across all reported metrics, and that pairwise differences between models under rectangle prompting are statistically significant across all metrics. Detailed Holm–Bonferroni-adjusted *p*-values are reported in Table 5.

### 4.2. Comparison to a Classical Prompt-Only Baseline

To contextualize the zero-shot results, we evaluated a simple classical baseline that requires no learning: for rectangle prompts, the prompt bounding box itself was treated as a filled segmentation mask (all pixels inside the box were labeled as vertebra), without using any image content. Baseline results are reported in Table 4 for a direct comparison with model-based zero-shot segmentation. Because our rectangle prompts are generated as tight boxes around the reference masks, this baseline is optimistic; however, it provides a transparent reference for what can be achieved using coarse localization alone. Notably, the baseline performs well for very tight boxes, but degrades for looser prompts; in the loosest box quartile, SAM-ViT-Huge improves IoU from 0.590 (box baseline) to 0.661 (model-score selection), indicating added value when prompt localization is imperfect.

### 4.3. Sensitivity to Bounding-Box Tightness

**Figure 2:**
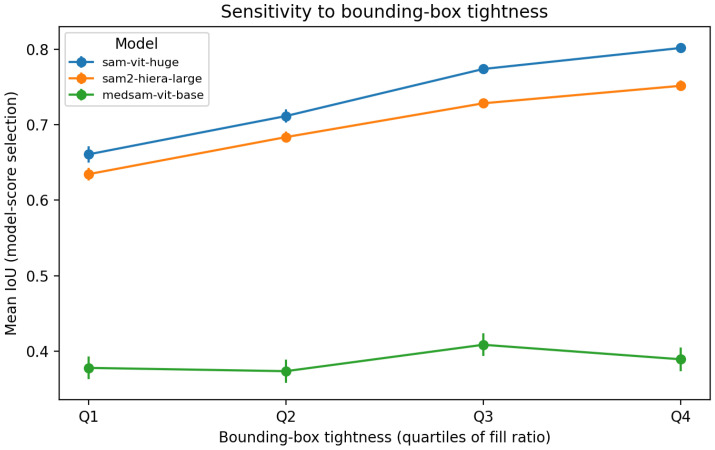
Mean IoU (model-score selection) as a function of bounding-box tightness quartiles. Tightness is measured by the fill ratio $r=A_{\mathrm{GT}}/A_{\mathrm{box}}$ (higher is tighter). Error bars show the standard error of the mean.

Rectangle prompts were generated as tight bounding boxes around the reference masks. To probe robustness to imperfect box prompts without requiring additional manual annotations, we quantified an effective box tightness as the fill ratio $r=A_{\mathrm{GT}}/A_{\mathrm{box}}$, where $A_{\mathrm{GT}}$ is the ground-truth mask area and $A_{\mathrm{box}}$ is the prompt box area. Lower *r* implies that the box contains proportionally more background and adjacent anatomy, which mimics a common clinical deviation where a user draws a looser box around the target. We stratified all vertebral instances into quartiles of *r* and evaluated performance using model-score-based mask selection (Table 4). As shown in Table 6 and Figure 2, SAM-ViT-Huge and SAM2-Hiera-Large exhibit a monotonic performance drop for looser boxes: mean IoU decreases from 0.802 to 0.661 (SAM-ViT-Huge) and from 0.751 to 0.634 (SAM2) between the tightest and loosest quartiles. This trend suggests that the amount of irrelevant context enclosed by a box prompt can materially affect segmentation quality, consistent with prior reports of prompt sensitivity in SAM-like models [81,82].

### 4.4. Image-Level Distributions, Uncertainty and Metric Relationships

While Table 3 reports instance-level mean performance aggregated over vertebral masks, an image-level analysis provides an additional view on robustness across radiographs. Specifically, each radiograph yields multiple vertebral instances (median 10) and failure cases may be concentrated in a subset of radiographs (e.g., severe curvature, low contrast, or partial field-of-view). Therefore, we aggregated vertebra-level metrics within each radiograph to obtain one score per image and visualized the resulting distributions.

#### 4.4.1. Overlap Metrics (Dice and IoU) Across Radiographs

**Figure 3:**
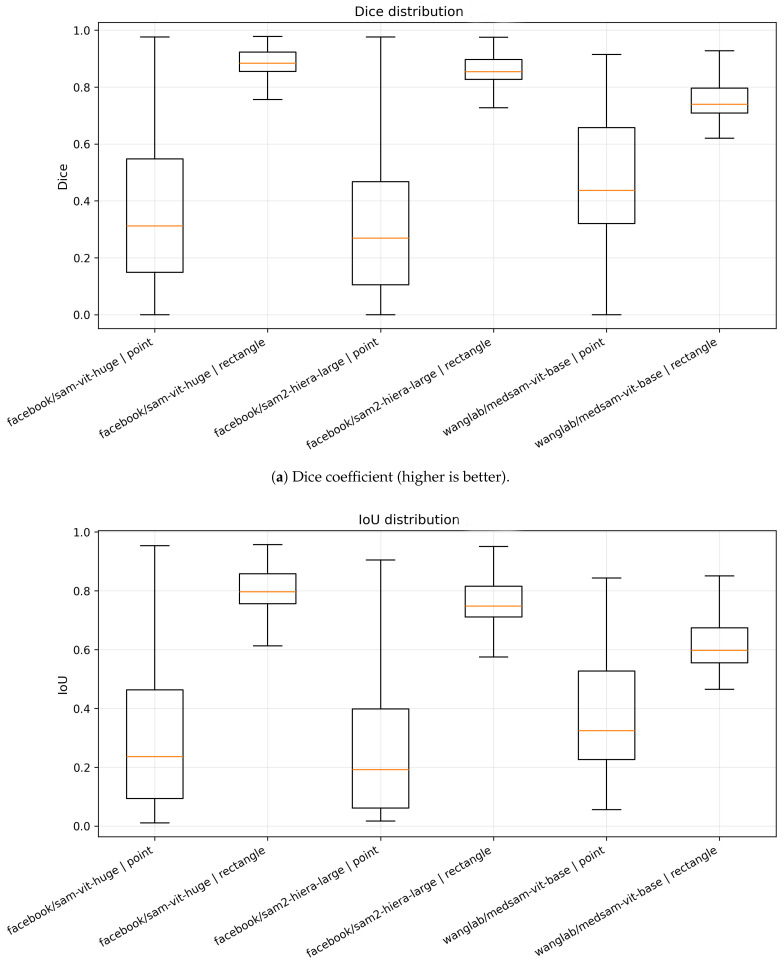
Image-level (radiograph-level) distribution of overlap metrics for all evaluated backbones and prompting strategies. For each radiograph, per-vertebra scores were averaged to produce a single image-level score. Boxes indicate the interquartile range (IQR), center lines denote the median and whiskers extend to 1.5 × IQR. Rectangle prompting yields consistently higher overlap and reduced dispersion compared with single-point prompting.

Figure 3 summarizes the image-level distributions of Dice and IoU for all model–prompt combinations. A consistent pattern emerges: rectangle prompting produces both higher overlap and tighter distributions than point prompting. For sam-vit-huge and sam2-hiera-large, rectangle prompts yield high medians (Dice∼0.86–0.90, IoU∼0.74–0.80) with relatively narrow interquartile ranges (IQRs), indicating stable behavior across radiographs. In contrast, point prompting results in substantially lower medians (Dice∼0.27–0.44, IoU∼0.19–0.32) and markedly broader IQRs, with whiskers extending toward both near-zero overlap (complete failures) and near-perfect overlap (isolated successes). This wide dispersion suggests that a single centroid point is frequently underconstraining in spine radiographs, where multiple anatomically similar vertebrae coexist in close proximity.

MedSAM exhibits a qualitatively similar dependence on prompt type but with a different operating point. Under point prompting, medsam-vit-base shows a higher central tendency than the two SAM variants (median Dice∼0.44, median IoU∼0.32), indicating improved localization from a single point in a subset of cases. However, rectangle prompting remains superior and reduces dispersion, with medians shifting upward (Dice∼0.74, IoU∼0.60). Overall, the boxplots reinforce the conclusion that prompt informativeness is a dominant factor in zero-shot vertebral segmentation.

#### 4.4.2. Surface Distance Metrics (ASSD and HD95) Across Radiographs

**Figure 4:**
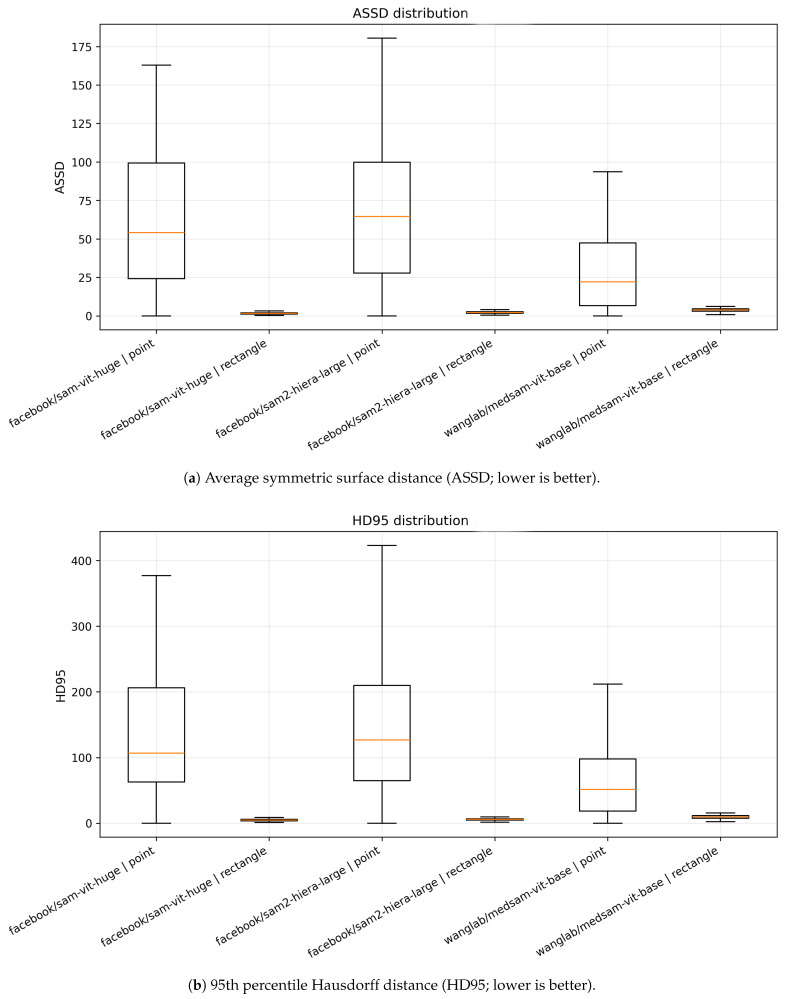
Image-level distribution of boundary-based metrics for all evaluated backbones and prompting strategies. Surface distances were computed from binary mask contours (Section 3.2.6) and are reported in millimeters when DICOM pixel spacing was available, otherwise in pixels. Rectangle prompting produces substantially smaller distances and tighter distributions, whereas point prompting results in large and heavy-tailed boundary errors.

To complement overlap measures, Figure 4 reports image-level surface distances, where lower values indicate better boundary alignment. Under rectangle prompting, all methods yield low distances with compact distributions, typically on the order of only a few pixels (or millimeters if pixel spacing was available), consistent with accurate boundary placement. For example, median ASSD values are ∼2–4 and median HD95 values are ∼6–10 for rectangle prompts. Conversely, point prompting leads to a dramatic increase in surface distances and heavy-tailed distributions. Median ASSD rises to tens of pixels (∼22–65 depending on the model) and HD95 frequently exceeds ∼50–130, with extreme outliers indicating severe leakage into adjacent anatomy. The strong separation between point and rectangle prompting in boundary metrics corroborates the overlap-based findings, but additionally highlights that point-based failures often correspond to clinically substantial contour misalignment.

#### 4.4.3. Mean Performance with Uncertainty Estimates

**Figure 5:**
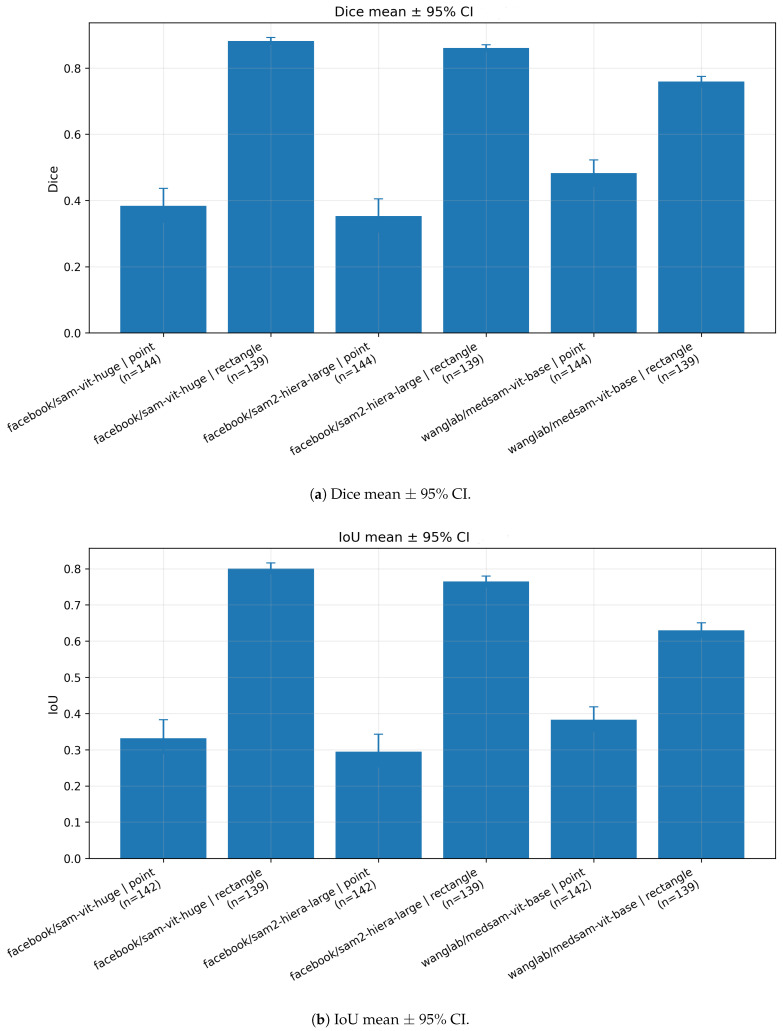
Mean image-level Dice and IoU with 95% bootstrap confidence intervals (CIs) computed over radiographs. The number of radiographs included for each configuration is shown in parentheses under the x-axis labels. Across all backbones, rectangle prompting yields higher mean overlap and smaller uncertainty than point prompting.

Figure 5 reports mean Dice and IoU at the image level with 95% bootstrap CIs. The uncertainty intervals are narrowest for rectangle prompting in sam-vit-huge and sam2-hiera-large, reflecting consistent performance across radiographs. In contrast, point prompting yields broader intervals, consistent with the larger dispersion observed in the boxplots. Across all backbones, the mean improvement from point to rectangle prompting is larger than the corresponding CI widths, indicating a robust and practically meaningful effect of prompt type.

#### 4.4.4. Global Score Distribution and Metric Redundancy

**Figure 6:**
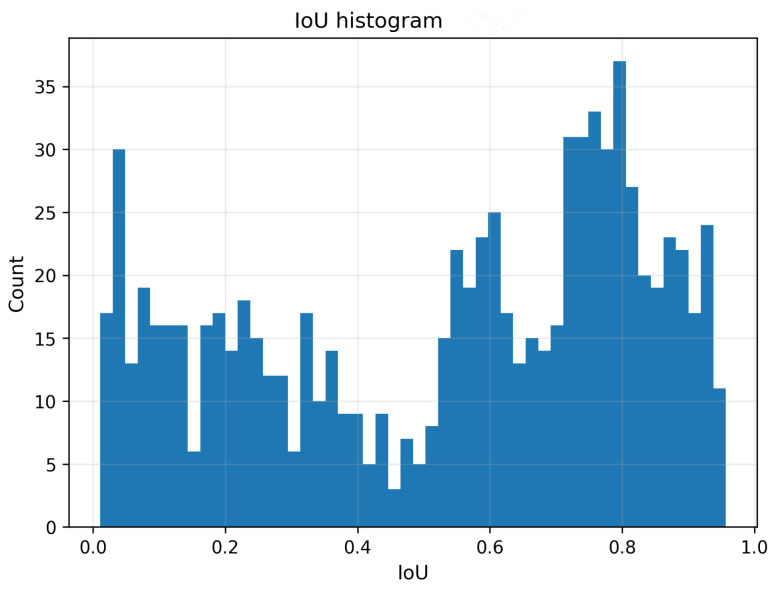
Histogram of image-level IoU values pooled across all evaluated configurations. The bimodal distribution reflects two distinct performance regimes associated with point prompts (low IoU) and rectangle prompts (high IoU).

**Figure 7:**
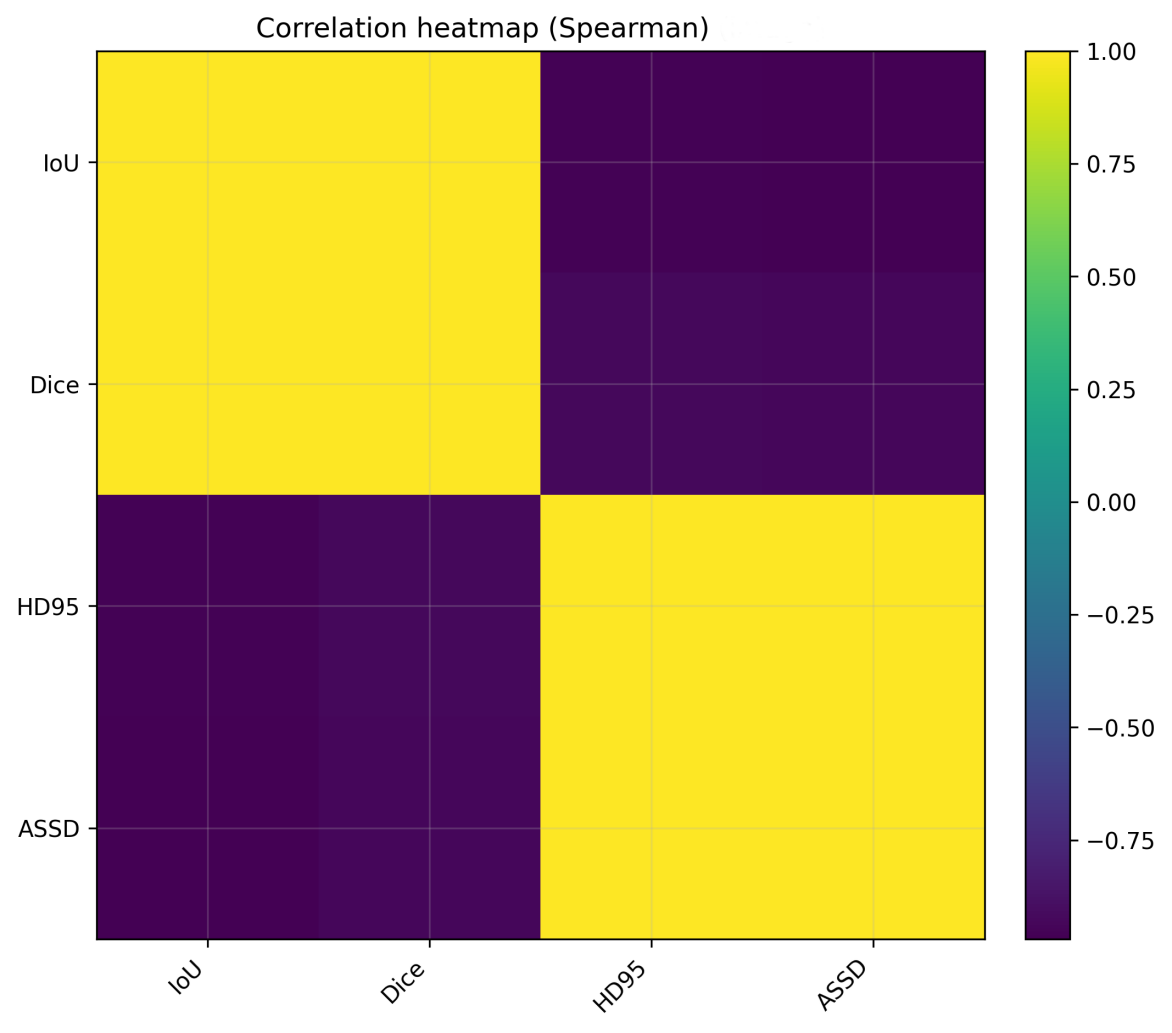
Spearman rank correlation matrix between overlap-based (IoU, Dice) and boundary-based (ASSD, HD95) metrics, computed on pooled image-level samples across all configurations. IoU and Dice are nearly perfectly correlated, ASSD and HD95 are strongly positively correlated and overlap metrics are strongly negatively correlated with surface distances.

Figure 6 shows the histogram of image-level IoU values pooled across all model–prompt configurations. The distribution is distinctly bimodal, with one mode at low IoU values (dominated by point prompting) and another at high IoU values (dominated by rectangle prompting), illustrating that the prompting strategy induces two clearly separated performance regimes. Finally, Figure 7 presents Spearman rank correlations among IoU, Dice, ASSD and HD95. IoU and Dice exhibit an almost perfect positive monotonic association, reflecting their deterministic relationship. Similarly, ASSD and HD95 are strongly positively correlated, indicating that both quantify related aspects of boundary error. Importantly, overlap-based metrics are strongly negatively correlated with surface distances, confirming that improved overlap is accompanied by reduced boundary discrepancies.

### 4.5. Failure Case Analysis and Point-Prompt Diagnostics

While mean overlap metrics summarize average segmentation quality, clinical deployment additionally requires understanding how often a configuration fails catastrophically and what those failures look like. Here we provide a focused failure-case analysis, with particular emphasis on single-point prompting, which showed the largest dispersion and heavy-tailed boundary errors in Figure 3 and Figure 4.

We operationally define an instance-level failure as IoU < 0.1 under the model-score selection protocol (i.e., selecting the candidate mask with the highest predicted IoU), because this protocol most closely reflects real-world zero-shot usage when ground truth is unavailable at inference time. To quantify boundary-error outliers, we additionally report the median and 95th percentile of the 95th-percentile Hausdorff distance (HD95) computed in millimeters for instances with available DICOM pixel spacing.

As shown in Table 7, point prompting fails frequently in a deployable setting: the failure rate (IoU < 0.1) reaches 81.6% for SAM-ViT-Huge and 81.1% for SAM2-Hiera-Large and remains high at 51.8% for MedSAM-ViT-Base. Rectangle prompting is substantially more reliable for the two SAM variants (<1% failures), whereas MedSAM-ViT-Base exhibits a higher failure frequency under model-score selection, consistent with its larger oracle-to-model-score gap. Boundary diagnostics corroborate that point prompting produces clinically large contour deviations: median HD95 exceeds 155 mm for the SAM variants under point prompts, compared with 4–6 mm under rectangle prompts.

**Figure 8:**
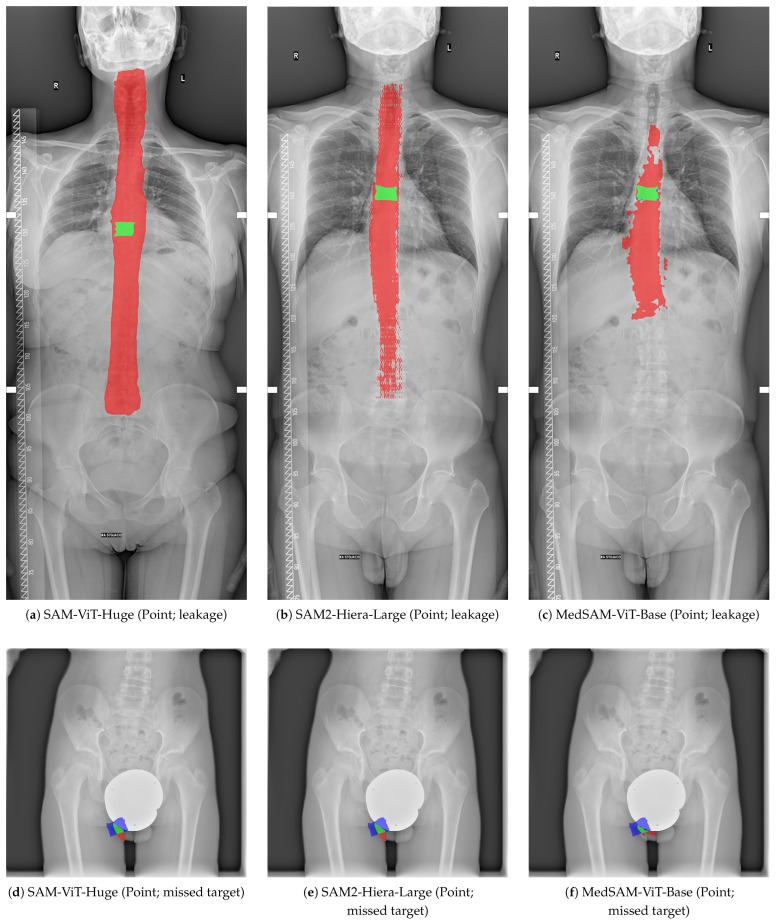
Representative point-prompt failure cases for all three evaluated backbones. (**Top**) row: leakage/over-segmentation with large false-positive spillover into adjacent anatomy (red) and correspondingly large boundary errors. (**Bottom**) row: missed-target/under-segmentation with substantial false negatives (blue). In all panels, green denotes true-positive overlap.

Inspection of the lowest-IoU point-prompt cases revealed two recurring failure modes: (i) leakage/over-segmentation, where the predicted mask spills into adjacent vertebrae and surrounding anatomy (high recall, very low precision and a large predicted-to-ground-truth area ratio), and (ii) missed-target/under-segmentation, where only a fragment of the vertebra is captured (low recall and an area ratio below one). Figure 8 visualizes representative examples of both modes for each backbone using difference overlays (green: true positives; red: false positives; blue: false negatives).

These diagnostics suggest that, in addition to overlap scores, simple sanity checks such as predicted area ratios and boundary-distance outlier thresholds could serve as lightweight quality-control indicators when deploying interactive zero-shot segmentation on multi-vertebra radiographs.

Representative failure cases for point-prompt segmentation are illustrated in Figure 8. These examples highlight two common error modes: leakage and missed targets. Representative boundary error diagnostics for point-prompt failure cases are summarized in Table 8.

### 4.6. Computational Efficiency and Inference Latency

In addition to accuracy, computational efficiency and inference latency are important for clinical usability. Table 9 summarizes wall-clock runtime for the standardized evaluation pipeline across models and prompt types. Across configurations, processing a single radiograph (containing on average about nine vertebral instances) required approximately 16–20 s, corresponding to about 1.8–2.2 s per vertebra. Among the tested backbones, SAM2-Hiera-Large achieved the lowest latency, whereas SAM-ViT-Huge was the slowest; nevertheless, the gap between the fastest and slowest rectangle configuration was modest (about 1.25×). Because the additional computation required for oracle vs. model-score mask selection is negligible compared to encoder/decoder inference, these latency figures apply to both selection protocols.

## 5. Discussion

### 5.1. Principal Findings and Overall Interpretation

This study investigated whether promptable segmentation foundation models can generalize zero-shot to vertebral instance segmentation on DICOM spine radiographs, without any task-specific fine-tuning. The results demonstrate three consistent and clinically relevant observations.

First, prompt informativeness dominated performance. This dependence on prompt informativeness is consistent with systematic evaluations of SAM-style models in medical imaging, where box-based prompts often yield more stable and anatomically constrained masks than sparse point prompts in crowded or low-contrast scenes. [6,12,47,48]. Across all evaluated backbones, rectangle (bounding-box) prompts substantially outperformed single-point prompts in overlap metrics (IoU, Dice), agreement measures (MCC, Cohen’s κ) and boundary-based distances (ASSD, HD95). In Table 3, the best-performing configuration was sam-vit-huge with rectangle prompting (IoU = 0.782, Dice = 0.870), followed closely by sam2-hiera-large with rectangle prompting (IoU = 0.744, Dice = 0.848). In contrast, all point-prompted configurations produced low overlap (IoU ≤0.319, Dice ≤0.414), despite often exhibiting high recall. This indicates that, on radiographs with multiple similar anatomical instances, a single point is frequently insufficient to unambiguously specify the intended vertebra.

Second, the failure modes under point prompting were systematic and severe. Point prompts yielded a characteristic pattern of very high recall but low precision (e.g., sam-vit-huge point: Precision = 0.293, Recall = 0.911), which is consistent with over-segmentation and leakage into surrounding anatomy. Similar ambiguity-driven leakage failures have been reported when SAM is applied to multi-instance or visually repetitive targets, where sparse prompts underconstrain the output and the model expands to adjacent structures [6,7,81]. Conversely, several clinical-oriented studies report that single-point or sparse prompting can be effective for isolated, visually distinctive targets (e.g., focal lesions) when combined with careful interaction design and quality control, highlighting that point prompting sensitivity is task- and modality-dependent. [8,40,41]. This behavior is further supported by the image-level distributions in Figure 3 and the heavy-tailed boundary errors in Figure 4, where point prompting led to large ASSD/HD95 values and extreme outliers, indicating clinically substantial contour deviations. The qualitative overlays in Figure 1 corroborate these quantitative trends by visualizing typical false-positive leakage patterns.

Third, model choice mattered, but less than prompt type. Among rectangle-prompted configurations, sam-vit-huge achieved the best overall overlap and agreement, while sam2-hiera-large showed slightly higher recall but somewhat lower precision, suggesting a more permissive segmentation behavior. medsam-vit-base performed worst under rectangle prompting (IoU = 0.599, Dice = 0.737) and also exhibited lower recall under point prompting (Recall = 0.762) than the two SAM variants. These differences suggest that, in this radiographic domain, backbone capacity and representation generality may outweigh the benefits of medical-domain adaptation learned from other modalities.

Taken together, the findings indicate that large promptable foundation models can produce accurate vertebral masks in spine radiographs in a zero-shot setting, but only when the prompt sufficiently constrains the spatial extent of the target instance.

### 5.2. Why Rectangle Prompts Work and Point Prompts Fail in Spine Radiographs

The strong dependence on prompt type is expected given the structure of spine radiographs and the nature of promptable segmentation. Radiographs contain multiple vertebrae that are highly repetitive in appearance and closely spaced, often with partial occlusions, low contrast boundaries and projection artifacts. In this setting, a single centroid point provides a weak constraint: it indicates where something should be segmented but not how far the object extends. A foundation model trained on broad visual data may then rely on global context and learned priors and can easily expand the mask to include adjacent vertebrae, the spinal column, ribs, or other high-contrast structures.

Rectangle prompts mitigate this ambiguity by explicitly specifying an approximate extent. This reduces the solution space and effectively suppresses competing hypotheses around the prompt location. The distributional results in Figure 3 highlight that rectangle prompts not only shift central tendency upward but also substantially reduce dispersion across radiographs. The histogram in Figure 6 further supports this interpretation: pooled IoU values form two distinct regimes, with low-overlap samples dominated by point prompting and high-overlap samples dominated by rectangle prompting. This bimodality suggests that the prompt type induces a qualitative transition in the model’s behavior, rather than merely a gradual improvement.

A practical implication is that interactive or semi-automatic workflows should favor bounding-box-based interactions, particularly in multi-instance radiographic settings. If point-based interaction is preferred (e.g., faster user input), it likely requires additional constraints such as multiple points, negative points, iterative refinement, or box+point hybrid prompting. These interaction patterns align with established interactive segmentation paradigms and recent error-tolerant frameworks that explicitly model imperfect clicks and iterative correction [48,83,84].

### 5.3. Model-Specific Behavior: SAM, SAM2 and MedSAM

Although prompt type was the main driver of performance, differences between backbones provide insight into generalization under domain shift.

#### 5.3.1. SAM-ViT-Huge

The strongest overall performance was achieved by sam-vit-huge under rectangle prompting (Table 3). This model also exhibited the highest precision among rectangle configurations, indicating that when spatial extent is constrained, the high-capacity image encoder and mask decoder can delineate vertebral boundaries reliably in most radiographs. The remaining errors likely arise from weak boundaries at vertebral endplates, partial visibility at the image margins and projection overlap with ribs and pelvis.

#### 5.3.2. SAM2-Hiera-Large

sam2-hiera-large performed competitively under rectangle prompting and achieved the highest recall (0.918). This suggests that SAM2 tends to include most vertebral pixels, but may be slightly more prone to over-coverage, consistent with its lower precision than sam-vit-huge. From an application standpoint, this trade-off may be acceptable in downstream pipelines that can tolerate mild over-segmentation but require high sensitivity (e.g., candidate region extraction followed by shape-based refinement). Recent evaluations of SAM2-style backbones in medical imaging similarly report competitive accuracy but note persistent challenges in low-contrast boundaries and dense-instance settings, which may manifest as slight over-coverage under weak constraints [10,35].

#### 5.3.3. MedSAM-ViT-Base

Despite being fine-tuned on large-scale medical segmentation data, medsam-vit-base did not outperform the two general-purpose SAM variants on radiographs. While medical-domain adaptation has been shown to improve robustness in several modalities and tasks, prior work also emphasizes that such gains can be modality-dependent and that prompt choice remains a major determinant of performance [6,9,69]. A plausible explanation is that MedSAM’s medical corpus may be dominated by modalities and tasks with boundary cues that differ substantially from projection radiography. Moreover, the ViT-Base backbone has lower capacity than ViT-H and the results suggest that capacity and general-purpose representations are important for robust out-of-domain generalization in complex radiographs. Interestingly, MedSAM showed relatively better point-prompt performance (higher mean IoU/Dice than the other models under point prompting), which may reflect stronger medical priors for objectness around localized prompts; however, these priors are still insufficient to reliably disambiguate vertebrae without explicit extent information.

### 5.4. Boundary Metrics and the Importance of Complementary Evaluation

Overlap metrics (IoU and Dice) capture region agreement but can underrepresent clinically relevant boundary deviations, particularly when masks are systematically larger than ground truth. Boundary-based distances (ASSD, HD95) provided a crucial complementary perspective in this study. This is consistent with established recommendations in medical image segmentation to report both region overlap and boundary distances, because overlap alone may obscure clinically meaningful contour deviations, especially for systematically over- or under-segmented masks [73].

The point-prompt configurations exhibited large ASSD and HD95 values with heavy tails (Figure 4), indicating frequent catastrophic boundary errors. These failures are consistent with leakage into adjacent vertebrae and surrounding anatomy, which may still produce moderate recall but are unacceptable for precise anatomical measurements. In contrast, rectangle prompting achieved compact boundary distance distributions, supporting the interpretation that the masks are not only overlapping but also geometrically aligned.

The correlation analysis in Figure 7 further clarifies metric redundancy and complementarity. IoU and Dice were nearly perfectly correlated, which is expected given their deterministic relationship. Similarly, ASSD and HD95 were strongly correlated, indicating that both quantify related aspects of contour error. Importantly, overlap metrics were strongly negatively correlated with boundary distances, confirming that improving overlap generally corresponds to improved boundary alignment in this benchmark. From a reporting standpoint, these results suggest that a minimal yet informative metric set could include one overlap metric (IoU or Dice) and one boundary metric (ASSD or HD95), with the remaining metrics used for additional interpretability (precision–recall balance and agreement).

### 5.5. Implications for Spinal Parameters Assessment Workflows

Accurate vertebral segmentation can support several steps in spinal parameters analysis, including vertebral localization, indexing and geometric measurement. In clinical practice, spine disorders severity is commonly assessed using angle measurements (e.g., Cobb angle), which require identification of end vertebrae and estimation of vertebral orientation. While the present study did not evaluate downstream clinical endpoints, the achieved overlap and boundary accuracy under rectangle prompting suggests that promptable segmentation models could serve as a pre-segmentation tool in semi-automatic pipelines.

A feasible workflow is human-in-the-loop: a clinician (or a pre-processing detector) provides a coarse bounding box for a target vertebra, the model outputs a mask and the mask is accepted or iteratively refined. This could reduce annotation time for dataset creation, accelerate semi-automatic measurement tools and enable rapid prototyping in settings where training data are limited. Related clinically oriented studies of SAM-style segmentation similarly emphasize human-in-the-loop prompting with quality control as a practical way to leverage strong foundation models despite imperfect zero-shot behavior [8,40]. For radiography-specific spine imaging, early work—including foundation-model-based studies and dedicated supervised pipelines—supports feasibility but highlights the importance of robust prompting and validation under acquisition variability [5,46]. However, for full automation, additional components are required: vertebra detection, vertebra indexing (T1–L5), endplate line estimation and quality control for failure cases.

From a computational perspective, the standardized pipeline achieved end-to-end runtimes on the order of tens of seconds per radiograph on a single CUDA-enabled GPU (Table 9). In particular, rectangle prompting required 16.15–20.15 s per radiograph across models, while per-vertebra latency was 1.79–2.24 s. This suggests that, once prompts are provided, promptable foundation models can return vertebral masks within time budgets compatible with semi-automatic measurement workflows, although implementation-level optimizations (e.g., batching and mixed precision) could further reduce latency.

Importantly, the point-prompt results indicate that extremely lightweight interactions (single clicks) are unlikely to be robust enough for multi-vertebra radiographs without additional constraints. Thus, the practical value of zero-shot SAM-like models in radiography may depend on either bounding-box prompts or multi-point iterative prompting, rather than single-point prompting.

### 5.6. Future Directions

Several directions could improve both performance and clinical utility.

#### 5.6.1. More Realistic Prompting and Interaction

A systematic evaluation with perturbed boxes, off-center points, multi-point prompts and negative points would quantify real-world robustness. Iterative prompting strategies (e.g., initial box followed by corrective clicks) could address failure cases while maintaining minimal interaction burden.

#### 5.6.2. Automatic Prompt Generation

To move toward automation, bounding boxes could be generated by a lightweight vertebra detector or a spine-localization module. Even coarse automatic boxes may be sufficient given the strong benefit observed for rectangle prompting, especially if coupled with simple post-processing (connected component filtering and shape regularization).

#### 5.6.3. Mask Selection Without Ground Truth

Because multi-mask output is inherent to SAM-like models, selecting the correct candidate mask is critical. Evaluating model-score-based selection versus oracle selection should be reported explicitly and improved selection heuristics (e.g., mask compactness priors, anatomical plausibility constraints, or consistency across adjacent vertebrae) could narrow the gap between upper-bound and deployable performance.

#### 5.6.4. Domain Adaptation and Lightweight Fine-Tuning

While the present study focused on strict zero-shot evaluation, lightweight adaptation strategies (e.g., adapters, LoRA, or prompt tuning) using a small labeled subset may yield substantial gains, particularly for point prompting and difficult boundary cases. Such adaptation should be evaluated under realistic annotation budgets and across institutions.

#### 5.6.5. Downstream Spinal Parameters Measurements

Finally, segmentation should be integrated into a full spinal parameters assessment pipeline to measure clinical endpoints and reliability. This includes vertebra indexing, endplate detection and automated angle computation with uncertainty estimation and quality control.

### 5.7. Summary

In a strictly zero-shot setting, promptable segmentation foundation models can achieve strong vertebral segmentation performance on DICOM spine radiographs, but performance is highly sensitive to prompt type. Bounding-box prompts enable stable, high-overlap, low-boundary-error segmentations across radiographs, whereas single-point prompts frequently lead to over-segmentation and large boundary deviations. Among the evaluated backbones, sam-vit-huge with rectangle prompting provided the most favorable accuracy–robustness trade-off. These findings support the feasibility of using foundation models as interactive or semi-automatic components in spine imaging workflows, while emphasizing the need for realistic prompt evaluation, non-oracle mask selection, external validation and clinical endpoint studies before deployment.

### 5.8. Methodological Considerations and Limitations

The main methodological limitations and generalization considerations are summarized below. This study was designed as a pilot feasibility benchmark and several limitations should be considered when interpreting the results.

#### 5.8.1. Oracle Mask Selection Inflates Performance Relative to Deployment

All models produced multiple candidate masks per prompt. The main evaluation selected the best mask by maximizing IoU against ground truth (oracle protocol), which estimates an upper bound on achievable performance given the candidate set. In real deployment, ground truth is not available and mask selection must rely on model-provided confidence scores or additional heuristics. Therefore, the reported results likely overestimate real-world performance unless a reliable selection strategy is implemented and validated. Notably, the original SAM design outputs a per-mask predicted quality score (often interpreted as an estimated IoU), which can serve as a deployable heuristic for candidate selection [68].

#### 5.8.2. Prompts Were Derived from Ground Truth and Do Not Capture Clinical Prompt Noise

Both rectangle prompts (tight bounding boxes) and point prompts (centroids) were generated from reference masks to ensure reproducibility and to isolate model behaviour from inter-operator variability. In clinical practice, user-provided boxes are rarely perfectly localized: boxes may be looser, may include parts of adjacent vertebrae, and may be shifted or truncated depending on the operator and the viewing conditions. To partially address this limitation, we added a sensitivity analysis that stratifies instances by the ground-truth-to-box area ratio (fill ratio $r=A_{\mathrm{GT}}/A_{\mathrm{box}}$; Section 4.3), which controls how much background/adjacent anatomy is included within the box even under a tight construction. We observed a monotonic drop in performance for SAM-ViT-Huge and SAM2-Hiera-Large when the effective box becomes looser (Table 6), indicating that segmentation quality is sensitive to the amount of irrelevant context enclosed by the prompt. Nevertheless, this proxy does not explicitly model box mis-centering or under-coverage; prior work has shown that SAM-like models can exhibit large output variations under small prompt perturbations and substantial inter-user variability in bounding boxes [81,82]. Several lines of work aim to mitigate prompt noise, including training or fine-tuning with perturbed bounding-box prompts [85], decoupling prompt quality from mask generation in SAM-like architectures [86], and designing error-tolerant interactive frameworks that explicitly account for imperfect user inputs [83,84]. These approaches are complementary and motivate future studies with manual prompts and controlled box perturbations in radiographic workflows.

#### 5.8.3. Intensity Normalization Without Modality-Specific Windowing

Radiographs were normalized using per-image min–max scaling after DICOM decoding and photometric correction, without applying DICOM VOI LUT or WindowCenter/WindowWidth-based intensity windowing. This choice avoids dependence on vendor-specific display presets and maintains a fully reproducible, model-agnostic pipeline, but it may under-utilize clinically tuned bone-contrast settings in some acquisitions. Future work will investigate windowing strategies (e.g., DICOM VOI LUT/WindowCenter/WindowWidth when reliably available, or learned normalization) and quantify their impact on segmentation accuracy and prompt robustness.

#### 5.8.4. Acquisition Variability and Radiography-Specific Intensity Non-Standardization

Projection radiography exhibits substantial variability across acquisition settings (e.g., view position, collimation, and dose), detector characteristics, and vendor post-processing pipelines (contrast enhancement and edge sharpening). Because radiograph pixel values are typically not calibrated to a standardized physical unit (unlike Hounsfield units in CT), such factors can alter global histograms and local contrast even after global normalization, and may therefore affect zero-shot generalization. Our study intentionally used a minimal, metadata-light DICOM preprocessing to remain reproducible and model-agnostic; however, future multi-institution evaluations should stratify performance by available acquisition metadata and test more robust intensity harmonization (e.g., VOI LUT when reliable, percentile-based scaling, or histogram standardization) to better control acquisition-driven variability.

#### 5.8.5. Single-Center Dataset, Domain Shift and Limited External Validation

The dataset contained 144 radiographs with 1309 annotated vertebrae. All images were collected within a single clinical environment, which likely limits the diversity of scanners/detectors and vendor post-processing pipelines represented in this benchmark. Although this enables robust instance-level evaluation, generalization to different hospitals, imaging devices, acquisition protocols, patient populations and spine disorder severities remains untested. In particular, domain shift can arise from differences in X-ray systems (detector response, resolution, noise characteristics and scatter management), protocol parameters (dose/exposure, collimation and view positioning) and proprietary enhancement (edge sharpening, contrast equalization), which may affect both the appearance of vertebral boundaries and the reliability of prompt-driven segmentation. Furthermore, patient anatomy diversity (age-related morphology, body habitus, congenital variants such as transitional vertebrae, severe deformities, fractures/degenerative changes, and post-operative instrumentation) may introduce shapes, occlusions and artifacts that are underrepresented in small single-center cohorts and can challenge zero-shot generalization. External validation on multi-institution cohorts is necessary before clinical conclusions can be drawn. Multi-center vertebra segmentation benchmarks in CT/MRI have shown that performance can vary across scanners and patient cohorts, underscoring the importance of external validation and stratified reporting [2,3]. Future work should therefore evaluate cross-site performance, stratify results by available scanner/protocol metadata, and assess robustness in challenging anatomical subgroups.

#### 5.8.6. Two-Dimensional Projection Challenges

Radiographs are 2D projections of a 3D structure. Overlaps, foreshortening and non-uniform contrast can make vertebral boundaries ambiguous even for human annotators. Some segmentation errors may therefore reflect inherent image ambiguity rather than model failure alone. In addition, left–right symmetry and repetitive anatomy can yield plausible but wrong segmentations under weak prompts.

#### 5.8.7. Clinical Endpoint Not Evaluated

This benchmark quantified segmentation agreement but did not assess the impact on spine-specific clinical measures (e.g., vertebral tilt, Cobb angle). A small boundary error may or may not translate into clinically meaningful measurement error depending on where it occurs (endplates vs. lateral borders). Future work should connect segmentation quality to measurement accuracy and inter-/intra-rater reliability.

## 6. Conclusions

This study assessed the feasibility of zero-shot vertebral instance segmentation on DICOM spine radiographs using promptable foundation models. Three backbones were evaluated under a fully standardized and reproducible pipeline (identical pre-processing, prompt generation, multi-mask handling and metric computation): facebook/sam-vit-huge, facebook/sam2-hiera-large and wanglab/medsam-vit-base. Performance was quantified on 144 radiographs with 1309 annotated vertebral masks using overlap-based (IoU, Dice) and boundary-based (ASSD, HD95) metrics.

The main conclusions are as follows: (i) Prompt type is the dominant factor in zero-shot vertebral segmentation. Bounding-box (rectangle) prompts consistently produced substantially higher overlap and markedly lower boundary errors than single-point prompts across all backbones. Point prompting typically resulted in over-segmentation (high recall but low precision) and heavy-tailed boundary deviations, indicating that a single localization cue is insufficient in radiographs containing many similar anatomical instances.

(ii) High-capacity generalist models perform best when provided with sufficiently informative prompts. Among the tested configurations, sam-vit-huge with rectangle prompting achieved the strongest overall performance (mean IoU = 0.782, Dice = 0.870), with sam2-hiera-large closely following (IoU = 0.744, Dice = 0.848) and exhibiting the highest recall under rectangle prompts (Recall = 0.918). medsam-vit-base improved substantially with rectangle prompts but remained below the two SAM variants (IoU = 0.599, Dice = 0.737), suggesting that, in this radiographic domain, backbone capacity and general visual representations are critical for robust transfer.

(iii) For practical use in spine disorders workflows, rectangle prompting is strongly recommended. The observed separation between point- and rectangle-based performance indicates that clinically useful segmentation quality in radiographs is achievable in a zero-shot regime primarily when the prompt constrains object extent. Therefore, the most realistic near-term application is a human-in-the-loop or semi-automatic workflow in which bounding boxes are provided by the user or generated automatically by a lightweight detector, followed by promptable mask generation.

Overall, the findings support the feasibility of using promptable foundation models for vertebral segmentation on DICOM radiographs in a strictly zero-shot setting, provided that prompts deliver sufficient spatial disambiguation.

## Figures and Tables

**Table 1:** Dataset composition and annotation density.

Characteristic	Value
Radiographs (unique DICOM images)	144
Annotated vertebrae (instance masks)	1309
Vertebrae per radiograph	mean 9.09 ± 4.26 (range 2–16)

**Table 2:** Native radiograph resolution and annotation size statistics.

Statistic	Radiograph Resolution (px)	Vertebra Mask Area (px^2^)
Mean ± SD	3404 ± 904 (height) 2575 ± 758 (width)	38,837 ± 28,449
Median [IQR]	3032 [3032–3667] (height) 3032 [1489–3032] (width)	32,277 [14,788–59,279]

**Table 3:** Quantitative segmentation performance under oracle mask selection for different models and prompt types (upper-bound evaluation). Values are reported as means with 95% bootstrap confidence intervals (shown in parentheses).

Model	Prompt	IoU	Dice	Precision	Recall	F1	MCC	Kappa
SAM-ViT-Huge	Point	0.265 (0.233–0.297)	0.311 (0.279–0.346)	0.293 (0.261–0.327)	0.911 (0.881–0.935)	0.311 (0.279–0.346)	0.380 (0.350–0.412)	0.306 (0.274–0.341)
SAM-ViT-Huge	Rectangle	0.782 (0.771–0.793)	0.870 (0.862–0.878)	0.850 (0.841–0.858)	0.906 (0.896–0.915)	0.870 (0.862–0.878)	0.874 (0.865–0.881)	0.870 (0.862–0.878)
SAM2-Hiera-Large	Point	0.224 (0.193–0.256)	0.276 (0.244–0.311)	0.249 (0.217–0.284)	0.907 (0.877–0.932)	0.276 (0.244–0.311)	0.354 (0.325–0.386)	0.271 (0.239–0.306)
SAM2-Hiera-Large	Rectangle	0.744 (0.734–0.755)	0.848 (0.841–0.855)	0.803 (0.793–0.813)	0.918 (0.910–0.924)	0.848 (0.841–0.855)	0.853 (0.847–0.860)	0.847 (0.840–0.854)
MedSAM-ViT-Base	Point	0.319 (0.296–0.344)	0.414 (0.387–0.444)	0.425 (0.392–0.460)	0.762 (0.735–0.787)	0.414 (0.387–0.444)	0.472 (0.448–0.499)	0.411 (0.384–0.441)
MedSAM-ViT-Base	Rectangle	0.599 (0.587–0.611)	0.737 (0.728–0.747)	0.729 (0.716–0.742)	0.781 (0.767–0.795)	0.737 (0.728–0.747)	0.745 (0.736–0.754)	0.736 (0.727–0.746)

**Table 4:** Quantitative segmentation performance under model-score-based (non-oracle, deployable) mask selection, including a prompt-only bounding-box baseline. For each prompt, the candidate mask with the highest model-provided confidence (predicted IoU) was selected. Values are reported as means with 95% bootstrap confidence intervals (shown in parentheses).

Model	Prompt	IoU	Dice	Precision	Recall	F1	MCC	Kappa
BBox-only baseline	Rectangle	0.731 (0.719–0.744)	0.841 (0.832–0.849)	0.731 (0.719–0.744)	1.000 (1.000–1.000)	0.841 (0.832–0.849)	0.852 (0.845–0.860)	0.840 (0.831–0.849)
SAM-ViT-Huge	Point	0.137 (0.112–0.165)	0.153 (0.127–0.181)	0.145 (0.119–0.173)	0.955 (0.926–0.978)	0.153 (0.127–0.181)	0.182 (0.154–0.211)	0.146 (0.120–0.175)
SAM-ViT-Huge	Rectangle	0.737 (0.723–0.750)	0.837 (0.827–0.848)	0.794 (0.781–0.808)	0.913 (0.900–0.926)	0.837 (0.827–0.848)	0.845 (0.835–0.854)	0.837 (0.826–0.847)
SAM2-Hiera-Large	Point	0.131 (0.105–0.161)	0.157 (0.128–0.188)	0.143 (0.114–0.175)	0.942 (0.913–0.965)	0.157 (0.128–0.188)	0.212 (0.183–0.243)	0.150 (0.122–0.182)
SAM2-Hiera-Large	Rectangle	0.699 (0.687–0.712)	0.815 (0.806–0.824)	0.730 (0.718–0.743)	0.949 (0.941–0.956)	0.815 (0.806–0.824)	0.826 (0.818–0.834)	0.814 (0.805–0.823)
MedSAM-ViT-Base	Point	0.222 (0.200–0.246)	0.293 (0.266–0.321)	0.340 (0.307–0.375)	0.724 (0.682–0.764)	0.293 (0.266–0.321)	0.356 (0.331–0.383)	0.289 (0.263–0.318)
MedSAM-ViT-Base	Rectangle	0.387 (0.363–0.412)	0.499 (0.470–0.528)	0.678 (0.656–0.701)	0.478 (0.447–0.508)	0.499 (0.470–0.528)	0.525 (0.498–0.552)	0.498 (0.469–0.527)

**Table 5:** Paired statistical comparisons across prompting strategies and models under model-score-based mask selection. Reported values are Holm–Bonferroni-adjusted *p*-values from two-sided paired Wilcoxon signed-rank tests computed on per-radiograph mean metrics.

Comparison	IoU	Dice	Precision	Recall	F1	MCC	Kappa
SAM-ViT-Huge: Rectangle vs. Point	6.20 × 10^−23^	6.20 × 10^−23^	6.20 × 10^−23^	1.63 × 10^−20^	6.20 × 10^−23^	6.20 × 10^−23^	6.20 × 10^−23^
SAM2-Hiera-Large: Rectangle vs. Point	6.20 × 10^−23^	6.20 × 10^−23^	2.41 × 10^−22^	2.65 × 10^−6^	6.20 × 10^−23^	6.20 × 10^−23^	6.20 × 10^−23^
MedSAM-ViT-Base: Rectangle vs. Point	4.79 × 10^−16^	5.25 × 10^−16^	1.90 × 10^−19^	7.06 × 10^−14^	5.25 × 10^−16^	5.95 × 10^−14^	4.26 × 10^−16^
SAM-ViT-Huge vs. SAM2-Hiera-Large (Rectangle)	7.48 × 10^−11^	1.38 × 10^−8^	3.69 × 10^−15^	8.50 × 10^−9^	1.38 × 10^−8^	2.16 × 10^−8^	1.38 × 10^−8^
SAM-ViT-Huge vs. MedSAM-ViT-Base (Rectangle)	6.20 × 10^−23^	6.20 × 10^−23^	3.73 × 10^−13^	6.20 × 10^−23^	6.20 × 10^−23^	6.20 × 10^−23^	6.20 × 10^−23^
SAM2-Hiera-Large vs. MedSAM-ViT-Base (Rectangle)	6.20 × 10^−23^	6.20 × 10^−23^	3.54 × 10^−4^	6.20 × 10^−23^	6.20 × 10^−23^	6.20 × 10^−23^	6.20 × 10^−23^

**Table 6:** Sensitivity analysis to bounding-box tightness for rectangle prompts using model-score-based mask selection. Instances are grouped by quartiles of the fill ratio $r=A_{\mathrm{GT}}/A_{\mathrm{box}}$; lower values correspond to looser boxes that include more background/adjacent anatomy within the prompt.

Model	Tightness Quartile	Fill Ratio Range	IoU	Dice
SAM-ViT-Huge	Q1 (loosest)	0.461–0.657	0.661	0.776
SAM-ViT-Huge	Q2	0.657–0.750	0.711	0.821
SAM-ViT-Huge	Q3	0.751–0.810	0.774	0.867
SAM-ViT-Huge	Q4 (tightest)	0.811–0.945	0.802	0.886
SAM-ViT-Huge	Δ (Q4–Q1)	–	0.141	0.109
SAM2-Hiera-Large	Q1 (loosest)	0.461–0.657	0.634	0.765
SAM2-Hiera-Large	Q2	0.657–0.750	0.684	0.805
SAM2-Hiera-Large	Q3	0.751–0.810	0.728	0.838
SAM2-Hiera-Large	Q4 (tightest)	0.811–0.945	0.751	0.852
SAM2-Hiera-Large	Δ (Q4–Q1)	–	0.117	0.086
MedSAM-ViT-Base	Q1 (loosest)	0.461–0.657	0.378	0.490
MedSAM-ViT-Base	Q2	0.657–0.750	0.374	0.483
MedSAM-ViT-Base	Q3	0.751–0.810	0.409	0.526
MedSAM-ViT-Base	Q4 (tightest)	0.811–0.945	0.389	0.499
MedSAM-ViT-Base	Δ (Q4–Q1)	–	0.011	0.010

**Table 7:** Failure rates under model-score-based mask selection and boundary-error quantiles under oracle selection. Failure rate is the percentage of vertebral instances with IoU below a threshold under model-score selection. Boundary distances (HD95) are computed in millimeters for instances with available DICOM pixel spacing.

Model	Prompt	Fail IoU < 0.1 (%)	Fail IoU < 0.2 (%)	Median HD95 (mm)	95th Pct HD95 (mm)
SAM-ViT-Huge	Point	81.6	82.5	155.1	400.4
SAM-ViT-Huge	Rectangle	0.7	1.1	4.3	11.0
SAM2-Hiera-Large	Point	81.1	83.3	167.7	421.9
SAM2-Hiera-Large	Rectangle	0.4	0.5	5.6	11.8
MedSAM-ViT-Base	Point	51.8	62.3	35.1	266.1
MedSAM-ViT-Base	Rectangle	23.4	30.3	9.7	18.1

**Table 8:** Boundary error diagnostics for the representative point-prompt failure cases shown in Figure 8. All values are reported under oracle mask selection; HD95 is in millimeters.

Failure Mode	Model	IoU	Precision	Recall	Area Ratio	HD95 (mm)
Leakage	MedSAM-ViT-Base	0.062	0.062	0.953	15.3	191.6
Leakage	SAM-ViT-Huge	0.005	0.005	1.000	198.3	662.3
Leakage	SAM2-Hiera-Large	0.044	0.044	0.999	22.9	327.9
Missed target	MedSAM-ViT-Base	0.141	0.529	0.161	0.3	14.5
Missed target	SAM-ViT-Huge	0.205	0.592	0.239	0.4	14.5
Missed target	SAM2-Hiera-Large	0.201	0.594	0.233	0.4	14.0

**Table 9:** Computational efficiency and inference latency measured as wall-clock time for the standardized evaluation pipeline. Reported times include image loading and pre-processing, per-radiograph embedding computation (cached), prompt encoding, mask decoding, post-processing and metric computation; export/plotting is excluded.

Model	Prompt	Radiographs	Instances	Total (min)	Time/Radiograph (s)	Time/Instance (s)
SAM-ViT-Huge	Rectangle	139	1253	46.7	20.15	2.24
SAM-ViT-Huge	Point	144	1309	42.9	17.86	1.96
MedSAM-ViT-Base	Rectangle	139	1253	38.9	16.79	1.86
MedSAM-ViT-Base	Point	144	1309	40.4	16.83	1.85
SAM2-Hiera-Large	Rectangle	139	1253	37.4	16.15	1.79
SAM2-Hiera-Large	Point	144	1309	39.4	16.43	1.81

## Data Availability

The original contributions presented in this study are included in the article. Further inquiries can be directed to the corresponding author.

## References

[1] Fatima J., Akram M.U., Jameel A., Syed A.M. (2021). Spinal vertebrae localization and analysis on disproportionality in curvature using radiography—A comprehensive review. Eurasip J. Image Video Process..

[2] Sekuboyina A., Husseini M.E., Bayat A., Löffler M., Liebl H., Li H., Tetteh G., Kukačka J., Payer C., Štern D. (2021). VERSE: A Vertebrae labelling and segmentation benchmark for multi-detector CT images. Med. Image Anal..

[3] Liebl H., Schinz D., Sekuboyina A., Malagutti L., Löffler M.T., Bayat A., El Husseini M., Tetteh G., Grau K., Niederreiter E. (2021). A computed tomography vertebral segmentation dataset with anatomical variations and multi-vendor scanner data. Sci. Data.

[4] Lessmann N., van Ginneken B., de Jong P.A., Išgum I. (2019). Iterative fully convolutional neural networks for automatic vertebra segmentation and identification. Med. Image Anal..

[5] Konya S., Sai Natarajan T., Allouch H., Nahleh K.A., Dogheim O.Y., Boehm H. (2021). Convolutional neural network-based automated segmentation and labeling of the lumbar spine X-ray. J. Craniovertebral Junction Spine.

[6] Mazurowski M.A., Dong H., Gu H., Yang J., Konz N., Zhang Y. (2023). Segment anything model for medical image analysis: An experimental study. Med. Image Anal..

[7] Mattjie C., de Moura L.V., Ravazio R., Kupssinskü L., Parraga O., Delucis M.M., Barros R.C. (2023). Zero-shot performance of the Segment Anything Model (SAM) in 2D medical imaging: A comprehensive evaluation and practical guidelines. Proceedings of the 2023 IEEE 23rd International Conference on Bioinformatics and Bioengineering (BIBE).

[8] Putz F., Beirami S., Schmidt M.A., May M.S., Grigo J., Weissmann T., Schubert P., Höfler D., Gomaa A., Hassen B.T. (2025). The Segment Anything foundation model achieves favorable brain tumor auto-segmentation accuracy in MRI to support radiotherapy treatment planning. Strahlenther. Und Onkol..

[9] Chang C., Law H., Poon C., Yen S., Lall K., Jamshidi A., Malis V., Hwang D., Bae W.C. (2025). Segment Anything Model (SAM) and Medical SAM (MedSAM) for Lumbar Spine MRI. Sensors.

[10] Dong H., Gu H., Chen Y., Yang J., Chen Y., Mazurowski M.A. (2026). Segment Anything Model 2: An Application to 2D and 3D Medical Images. IEEE Trans. Biomed. Eng..

[11] Khazanchi R., Govind S., Jain R., Du R., Dahdaleh N.S., Ahuja C.S., El Tecle N. (2025). Zero-shot segmentation of spinal vertebrae with metastatic lesions: An analysis of Meta’s Segment Anything Model 2 and factors affecting learning free segmentation. Neurosurg. Focus.

[12] Stein J., Di Folco M., Schnabel J.A. (2024). Influence of Prompting Strategies on Segment Anything Model (SAM) for Short-axis Cardiac MRI Segmentation. Proceedings of the German Conference on Medical Image Computing.

[13] Magg C., Verweij L.P., ter Wee M.A., Buijs G.S., Dobbe J.G., Streekstra G.J., Blankevoort L., Sánchez C.I. (2024). Training-free Prompt Placement by Propagation for SAM Predictions in 3D Bone CT Scans. Bildverarbeitung für die Medizin 2024.

[14] Fan D., Zhao J., Li C., Wang X., Zhang R., Zhu Q., Wang M., Si H., Zhang D., Sun L. (2025). MA-SAM: A Multi-Atlas Guided SAM Using Pseudo Mask Prompts Without Manual Annotation for Spine Image Segmentation. IEEE Trans. Med. Imaging.

[15] Wu Y., Wang Z., Yang X., Kang H., He A., Li T. (2025). Trans-SAM: Transfer Segment Anything Model to medical image segmentation with Parameter-Efficient Fine-Tuning. Knowl.-Based Syst..

[16] Ocepek D., Podobnik G., Ibragimov B., Vrtovec T. Deep Implicit Statistical Shape Models for 3D Lumbar Vertebrae Image Delineation. Proceedings of the Medical Imaging 2024: Image Processing.

[17] Kim H., Park J. Vertebral Segmentation without Training using Differentiable Appearance Modeling of a Deformable Spine Template. Proceedings of the Medical Imaging 2024: Image Processing.

[18] Hille G., Saalfeld S., Serowy S., Tönnies K. (2018). Vertebral body segmentation in wide range clinical routine spine MRI data. Comput. Methods Programs Biomed..

[19] Rak M., Steffen J., Meyer A., Hansen C., Tönnies K.D. (2019). Combining convolutional neural networks and star convex cuts for fast whole spine vertebra segmentation in MRI. Comput. Methods Programs Biomed..

[20] Masuzawa N., Kitamura Y., Nakamura K., Iizuka S., Simo-Serra E. (2020). Automatic Segmentation, Localization, and Identification of Vertebrae in 3D CT Images Using Cascaded Convolutional Neural Networks. Proceedings of the Medical Image Computing and Computer Assisted Intervention—MICCAI 2020.

[21] Altini N., De Giosa G., Fragasso N., Coscia C., Sibilano E., Prencipe B., Hussain S.M., Brunetti A., Buongiorno D., Guerriero A. (2021). Segmentation and identification of vertebrae in ct scans using cnn, k-means clustering and k-nn. Informatics.

[22] Möller H., Graf R., Schmitt J., Keinert B., Schön H., Atad M., Sekuboyina A., Streckenbach F., Kofler F., Kroencke T. (2025). SPINEPS—Automatic whole spine segmentation of T2-weighted MR images using a two-phase approach to multi-class semantic and instance segmentation. Eur. Radiol..

[23] Zhou Z., Zhang Z., Li M., Song L., Kong Y., Coatrieux J.L., Shu H. (2025). Mask prompt-guided multi-stage network for vertebrae identification. Biomed. Signal Process. Control.

[24] Mao Y., Feng Q., Zhang Y., Ning Z. (2025). Semantics and instance interactive learning for labeling and segmentation of vertebrae in CT images. Med. Image Anal..

[25] Lu H., Liu M., Yu K., Fang Y., Zhao J., Shi Y. (2025). A Deep Learning-Based Fully Automated Vertebra Segmentation and Labeling Workflow. Br. J. Hosp. Med..

[26] Hashia B., Mir A.H. (2020). Segmentation Techniques for the Diagnosis of Intervertebral Disc Diseases.

[27] Li Z., Zhou X., Tong T. (2024). A Two-Stage Network for Segmentation of Vertebrae and Intervertebral Discs: Integration of Efficient Local-Global Fusion Using 3D Transformer and 2D CNN. Proceedings of the Neural Information Processing.

[28] Harouni M., Goyal V., Feldman G., Michael S., Voss T.C. (2025). Deep Multi-Scale and Attention-Based Architectures for Semantic Segmentation in Biomedical Imaging. Comput. Mater. Contin..

[29] Liu X., Deng W., Liu Y. (2021). Application of hybrid network of unet and feature pyramid network in spine segmentation. Proceedings of the 2021 IEEE International Symposium on Medical Measurements and Applications (MeMeA).

[30] Stockton R., Albano J., Lentz J., Ganz M., Grewal K., Katsigiorgis G. (2019). A comparison of lumbar transverse pedicle angles between ethnic groups: A retrospective review. BMC Musculoskelet. Disord..

[31] Lepcha D.C., Goyal B., Dogra A., Alkhayyat A., Sahu P.K., Ali A., Kukreja V. (2025). Deep Learning in Medical Image Analysis: A Comprehensive Review of Algorithms, Trends, Applications, and Challenges. CMES—Comput. Model. Eng. Sci..

[32] Ma J., Wang A., Lin F., Wesarg S., Erdt M. (2019). A novel robust kernel principal component analysis for nonlinear statistical shape modeling from erroneous data. Comput. Med Imaging Graph..

[33] Chen Z., Qiu T., Huo L., Yu L., Shi H., Zhang Y., Wang H. (2019). Inter-Subject Shape Correspondence Computation from Medical Images without Organ Segmentation. IEEE Access.

[34] You X., Lou Y., Zhang M., Yang J., Gu Y. (2025). SLoRD: Structural Low-Rank Descriptors for Shape Consistency in Vertebrae Segmentation. IEEE J. Biomed. Health Inform..

[35] Blomenkamp L., Kramer I., Bauer S., Paulus D. (2025). A Novel Approach for Shape Segmentation of Vertebrae: Decomposition into Anatomical Regions Using 3D Skeletonization. Proceedings of the 2025 47th Annual International Conference of the IEEE Engineering in Medicine and Biology Society (EMBC).

[36] Zhang Z., Liu T., Fan G., Li N., Li B., Pu Y., Feng Q., Zhou S. (2025). SpineMamba: Enhancing 3D spinal segmentation in clinical imaging through residual visual Mamba layers and shape priors. Comput. Med. Imaging Graph..

[37] Wang H., Li Y., Fu Y., Lv C., Li F., Xi Y., Yang H. (2026). SPMamba: Spinal image segmentation via Mamba framework with hierarchical feature fusion and channel sorting. Biomed. Signal Process. Control.

[38] Zhao Y., Zhang J. (2024). Automatic Generation Method of Lumbar Spine Using Virtual Scoliosis Cases. Jisuanji Gongcheng/Computer Eng..

[39] Ding H., Seenivasan L., Killeen B.D., Cho S.M., Unberath M. (2024). Digital twins as a unifying framework for surgical data science: The enabling role of geometric scene understanding. Artif. Intell. Surg..

[40] Ali L., Alnajjar F., Swavaf M., Elharrouss O., Abd-alrazaq A., Damseh R. (2024). Evaluating segment anything model (SAM) on MRI scans of brain tumors. Sci. Rep..

[41] Deng R., Cui C., Liu Q., Yao T., Remedios L.W., Bao S., Landman B.A., Wheless L.E., Coburn L.A., Wilson K.T. (2025). Segment Anything Model (SAM) for Digital Pathology: Assess Zero-shot Segmentation on Whole Slide Imaging. Proceedings of the Electronic Imaging.

[42] Wang S., Bai Y. Enhancing the Medical Image Segmentation capability of Segment Anything Model. Proceedings of the ISAIMS 2024: 2024 5th International Symposium on Artificial Intelligence for Medicine Science.

[43] Nanni L., Fusaro D., Fantozzi C., Pretto A. (2023). Improving Existing Segmentators Performance with Zero-Shot Segmentators. Entropy.

[44] Sengupta S., Chakrabarty S., Soni R. Is SAM 2 Better than SAM in Medical Image Segmentation?. Proceedings of the Medical Imaging 2025: Image Processing.

[45] Yamagishi Y., Hanaoka S., Kikuchi T., Nakao T., Nakamura Y., Nomura Y., Miki S., Yoshikawa T., Abe O. (2025). Using Segment Anything Model 2 for Zero-Shot 3D Segmentation of Abdominal Organs in Computed Tomography Scans to Adapt Video Tracking Capabilities for 3D Medical Imaging: Algorithm Development and Validation. JMIR AI.

[46] Simons S.J., Papiez B.W. (2025). SpineFM: Leveraging Foundation Models for Automatic Spine X-Ray Segmentation. Proceedings of the 2025 IEEE 22nd International Symposium on Biomedical Imaging (ISBI).

[47] Nouman M., Khoriba G., Rashed E.A. (2025). Evaluating Segmentation Accuracy with Diverse Prompt Strategies in Medsam. Proceedings of the 2025 IEEE 22nd International Symposium on Biomedical Imaging (ISBI).

[48] Ali H., Bulbul M.F., Shah Z. (2023). Prompt Engineering in Medical Image Segmentation: An Overview of the Paradigm Shift. Proceedings of the 2023 IEEE International Conference on Artificial Intelligence, Blockchain, and Internet of Things (AIBThings).

[49] Li C., Sultan R.I., Khanduri P., Qiang Y., Indrin C., Zhu D. (2025). AutoProSAM: Automated Prompting SAM for 3D Multi-Organ Segmentation. Proceedings of the 2025 IEEE/CVF Winter Conference on Applications of Computer Vision (WACV).

[50] Chen Y., Ivanova A., Saeed S.U., Hargunani R., Huang J., Liu C., Hu Y. (2024). Segmentation by Registration-Enabled SAM Prompt Engineering Using Five Reference Images. Proceedings of the 11th International Workshop, WBIR 2024, Held in Conjunction with MICCAI 2024.

[51] Liu X., Shi G., Wang R., Lai Y., Zhang J., Han W., Lei M., Li M., Zhou X., Wu Y. (2025). Segment Any Tissue: One-shot reference guided training-free automatic point prompting for medical image segmentation. Med. Image Anal..

[52] Gaus Y.F.A., Bhowmik N., Isaac-Medina B.K.S., Breckon T.P. (2024). Performance Evaluation of Segment Anything Model with Variational Prompting for Application to Non-Visible Spectrum Imagery. Proceedings of the 2024 IEEE/CVF Conference on Computer Vision and Pattern Recognition Workshops (CVPRW).

[53] Koleilat T., Asgariandehkordi H., Rivaz H., Xiao Y. (2024). MedCLIP-SAM: Bridging Text and Image Towards Universal Medical Image Segmentation. Proceedings of the Medical Image Computing and Computer Assisted Intervention—MICCAI 2024.

[54] Aleem S., Wang F., Maniparambil M., Arazo E., Dietlmeier J., Curran K., O’Connor N.E., Little S. (2024). Test-Time Adaptation with SaLIP: A Cascade of SAM and CLIP for Zero-shot Medical Image Segmentation. Proceedings of the 2024 IEEE/CVF Conference on Computer Vision and Pattern Recognition Workshops (CVPRW).

[55] Wu J., Xu M. (2024). One-Prompt to Segment All Medical Images. Proceedings of the 2024 IEEE/CVF Conference on Computer Vision and Pattern Recognition (CVPR).

[56] Shi W., He J., Shen Y. (2025). SIT-SAM: A semantic-integration transformer that adapts the Segment Anything Model to zero-shot medical image semantic segmentation. Biomed. Signal Process. Control.

[57] Liu X., Fu K., Jiang Y., Zhao Q. (2025). Promoting Segment Anything Model towards Highly Accurate Dichotomous Image Segmentation. Proceedings of the 2025 IEEE International Conference on Multimedia and Expo (ICME).

[58] Bui N.T., Hoang D.H., Tran M.T., Doretto G., Adjeroh D., Patel B., Choudhary A., Le N. (2024). SAM3D: Segment Anything Model in Volumetric Medical Images. Proceedings of the 2024 IEEE International Symposium on Biomedical Imaging (ISBI).

[59] Chen C., Miao J., Wu D., Zhong A., Yan Z., Kim S., Hu J., Liu Z., Sun L., Li X. (2024). MA-SAM: Modality-agnostic SAM adaptation for 3D medical image segmentation. Med. Image Anal..

[60] Wang H., Lin Y., Ding X., Li X. (2024). Tri-Plane Mamba: Efficiently Adapting Segment Anything Model for 3D Medical Images. Medical Image Computing and Computer Assisted Intervention—MICCAI 2024.

[61] Guo C., Jin Y., Tao B., Li J., Dai H.N., Li P. (2025). ZAP-2.5DSAM: Zero additional parameters advancing 2.5D SAM adaptation to 3D tumor segmentation. Vis. Comput..

[62] Dutt R., Ericsson L., Sanchez P., Tsaftaris S.A., Hospedales T. (2023). Parameter-Efficient Fine-Tuning for Medical Image Analysis: The Missed Opportunity. arXiv.

[63] Peng Z., Xu Z., Zeng Z., Xie L., Tian Q., Shen W. (2024). Parameter Efficient Fine-Tuning via Cross Block Orchestration for Segment Anything Model. Proceedings of the 2024 IEEE/CVF Conference on Computer Vision and Pattern Recognition (CVPR).

[64] Dhinagar N.J., Ozarkar S.S., Buwa K.U., Thomopoulos S.I., Owens-Walton C., Laltoo E., Jagad C., Chen Y.L., Cook P., McMillan C. Parameter Efficient Fine-tuning of Transformer-based Masked Autoencoder Enhances Resource Constrained Neuroimage Analysis. Proceedings of the Medical Imaging 2025: Computer-Aided Diagnosis.

[65] Sun G., Lei K., Li T., Yu L., Zhu S. (2026). Parameter-efficient fine-tuning for no-reference image quality assessment: Empirical studies on vision transformer. Displays.

[66] Peng F., Wang X. (2025). DABC-Net: A hierarchical deformation feature aggregation network with boundary-aware supervision for cardiac structure segmentation. Appl. Intell..

[67] Bidgood W.D., Horii S.C., Prior F.W., Van Syckle D.E. (1997). Understanding and Using DICOM, the Data Interchange Standard for Biomedical Imaging. J. Am. Med Inform. Assoc..

[68] Kirillov A., Mintun E., Ravi N., Mao H., Rolland C., Gustafson L., Xiao T., Whitehead S., Berg A.C., Lo W.Y. (2023). Segment Anything. Proceedings of the 2023 IEEE/CVF International Conference on Computer Vision (ICCV).

[69] Ma J., He Y., Li F., Han L., You C., Wang B. (2024). Segment anything in medical images. Nat. Commun..

[70] Ravi N., Gabeur V., Hu Y.T., Hu R., Ryali C., Ma T., Khedr H., Rädle R., Rolland C., Gustafson L. (2024). SAM 2: Segment Anything in Images and Videos. arXiv.

[71] Mason D. (2011). SU-E-T-33: Pydicom: An Open Source DICOM Library. Med. Phys..

[72] Hu M.K. (1962). Visual Pattern Recognition by Moment Invariants. IRE Trans. Inf. Theory.

[73] Taha A.A., Hanbury A. (2015). Metrics for evaluating 3D medical image segmentation: Analysis, selection, and tool. BMC Med. Imaging.

[74] Yeghiazaryan V., Voiculescu I. (2018). Family of boundary overlap metrics for the evaluation of medical image segmentation. J. Med. Imaging.

[75] Matthews B.W. (1975). Comparison of the predicted and observed secondary structure of T4 phage lysozyme. Biochim. Biophys. Acta-(BBA)–Protein Struct..

[76] Cohen J. (1960). A coefficient of agreement for nominal scales. Educ. Psychol. Meas..

[77] Maurer C.R., Qi R., Raghavan V. (2003). A linear time algorithm for computing exact Euclidean distance transforms of binary images in arbitrary dimensions. IEEE Trans. Pattern Anal. Mach. Intell..

[78] Huttenlocher D.P., Klanderman G.A., Rucklidge W.J. (1993). Comparing Images Using the Hausdorff Distance. IEEE Trans. Pattern Anal. Mach. Intell..

[79] Efron B. (1979). Bootstrap Methods: Another Look at the Jackknife. Ann. Stat..

[80] Wilcoxon F. (1945). Individual Comparisons by Ranking Methods. Biom. Bull..

[81] Li C., Huang Y., Li W., Liu H., Liu X., Xu Q., Chen Z., Huang Y., Yuan Y. Flaws can be Applause: Unleashing Potential of Segmenting Ambiguous Objects in SAM. Proceedings of the Advances in Neural Information Processing Systems (NeurIPS).

[82] Moskalenko A., Kuznetsov D., Dudko I., Iasakova A., Boldyrev N., Shepelev D., Spiridonov A., Kuznetsov A., Shakhuro V. (2026). BREPS: Bounding-Box Robustness Evaluation of Promptable Segmentation. arXiv.

[83] Gao Y., Sheng J., Wu W., Li H., Dong Y., Ge C., Yuan F., Gao X. (2025). SafeClick: Error-Tolerant Interactive Segmentation of Any Medical Volumes via Hierarchical Expert Consensus. arXiv.

[84] Zhang C., Liu T., Qu X., Liu L., Zhao Y., Wei Y. (2025). NTClick: Achieving Precise Interactive Segmentation With Noise-tolerant Clicks. Proceedings of the IEEE/CVF Conference on Computer Vision and Pattern Recognition (CVPR).

[85] Rahman M.M., Munir M., Jha D., Bagci U., Marculescu R. (2024). PP-SAM: Perturbed Prompts for Robust Adaptation of Segment Anything Model for Polyp Segmentation. arXiv.

[86] Gao Y., Xia W., Hu D., Wang W., Gao X. (2024). DeSAM: Decoupled Segment Anything Model for Generalizable Medical Image Segmentation. arXiv.

